# Molecular and proteomic analyses highlight the importance of ubiquitination for the stress resistance, metabolic adaptation, morphogenetic regulation and virulence of *Candida albicans*

**DOI:** 10.1111/j.1365-2958.2011.07542.x

**Published:** 2011-03

**Authors:** Michelle D Leach, David A Stead, Evelyn Argo, Donna M MacCallum, Alistair J P Brown

**Affiliations:** School of Medical Sciences, University of Aberdeen, Institute of Medical SciencesForesterhill, Aberdeen AB25 2ZD, UK

## Abstract

Post-translational modifications of proteins play key roles in eukaryotic growth, differentiation and environmental adaptation. In model systems the ubiquitination of specific proteins contributes to the control of cell cycle progression, stress adaptation and metabolic reprogramming. We have combined molecular, cellular and proteomic approaches to examine the roles of ubiquitination in *Candida albicans*, because little is known about ubiquitination in this major fungal pathogen of humans. Independent null (*ubi4/ubi4*) and conditional (*MET3p-UBI4/ubi4*) mutations were constructed at the *C. albicans* polyubiquitin-encoding locus. These mutants displayed morphological and cell cycle defects, as well as sensitivity to thermal, oxidative and cell wall stresses. Furthermore, *ubi4/ubi4* cells rapidly lost viability under starvation conditions. Consistent with these phenotypes, proteins with roles in stress responses (Gnd1, Pst2, Ssb1), metabolism (Acs2, Eno1, Fba1, Gpd2, Pdx3, Pgk1, Tkl1) and ubiquitination (Ubi4, Ubi3, Pre1, Pre3, Rpt5) were among the ubiquitination targets we identified, further indicating that ubiquitination plays key roles in growth, stress responses and metabolic adaptation in *C. albicans*. Clearly ubiquitination plays key roles in the regulation of fundamental cellular processes that underpin the pathogenicity of this medically important fungus. This was confirmed by the observation that the virulence of *C. albicans ubi4/ubi4* cells is significantly attenuated.

## Introduction

Post-translational modifications of proteins play critical roles in the cellular adaptation of all organisms, as well as their growth, division, differentiation and development. These modifications provide essential mechanisms by which the functions, activities and stabilities of pre-existing proteins can be rapidly and specifically modulated, thereby controlling dynamic cellular processes. Regulatory protein modifications include phosphorylation, acetylation, sumoylation and ubiquitination.

Ubiquitin is a highly conserved, small polypeptide of 76 amino acids found in all eukaryotic cells. The amino acid sequence of ubiquitin is identical across the animal kingdom, and differs by only three residues between yeast and animals (Ozkaynak *et al*., 1987; Finley and Chau, 1991). Within the cell, ubiquitin may be free or covalently attached to a range of cytoplasmic and nuclear proteins (Finley and Chau, 1991). Ubiquitin becomes covalently attached to free amino groups on target proteins through its carboxy-terminal glycine, a reaction catalysed by ubiquitin ligases (Jentsch *et al*., 1990; Stadtman, 1990; Rechsteiner, 1991). This enzymatic reaction is driven by an E1 ubiquitin-activating enzyme, an E2 ubiquitin-conjugating enzyme and an E3 ubiquitin ligase (Weissman, 2001).

The formation and processing of protein–ubiquitin conjugates, termed ‘ubiquitination’, is important for a number of biological processes. For example, ubiquitination plays a key role in the targeting of specific proteins for cytoplasmic degradation. Abnormal or short-lived proteins are extensively polyubiquitinated, consequently targeting their degradation by the multicatalytic 26S proteasome complex (Ciechanover, 1998; Ciechanover *et al*., 2000a,b;). The ubiquitin system also plays an important role in the degradation of denatured proteins following environmental stress (Finley *et al*., 1987; Amerik *et al*., 2005). The ubiquitination of some proteins is controlled in response to specific stimuli, thereby regulating their activities via relocalization and/or degradation (Finley and Chau, 1991; Paiva *et al*., 2009). Thus, ubiquitination is essential for the accurate execution of many cellular processes involving protein degradation, such as the cell cycle, endocytosis and the stress response (Hershko, 1997; Wolf and Hilt, 2004; Staub and Rotin, 2006).

The major opportunistic pathogen of humans, *Candida albicans*, remains one of the most persistent yeast pathogens known to man. It is a relatively harmless commensal of the oral cavity, gastrointestinal tract and genitalia of humans, but frequently causes superficial mucosal infections such as oral thrush and vaginitis in otherwise healthy individuals (Odds, 1988; Calderone, 2002). In the immunocompromised host, *C. albicans* can cause deep-seated systemic infections of the bloodstream and internal organs. Hence, *C. albicans* occupies a variety of niches within the human body, encountering a range of stressful conditions as it interacts with the host and its immunological defences (Lorenz *et al*., 2004; Fradin *et al*., 2005). For example, *C. albicans* encounters potentially damaging reactive oxidative species in some niches through the action of host defences (Enjalbert *et al*., 2007). In an attempt to counteract these defences, *C. albicans* has evolved effective oxidative stress responses, and these promote the survival of this pathogen in the host (Alonso-Monge *et al*., 1999; Hwang *et al*., 2002; Chauhan *et al*., 2006; Brown *et al*., 2009). In addition, evolutionarily conserved thermal adaptation mechanisms help *C. albicans* tune the levels of essential chaperones to the ambient temperature of host niches (Nicholls *et al*., 2009). Furthermore, *C. albicans* modulates its metabolism, cell morphology, stress responses and other cellular processes to the diverse microenvironments it encounters in the host (Hube, 1996; Ernst, 2000; Mavor *et al*., 2005; Kim and Kim, 2010). Therefore, a powerful combination of virulence factors and fitness attributes promote the virulence of this major pathogen. Protein phosphorylation has been shown to play critical roles in the regulation of many of these processes (Liu *et al*., 1994; Leberer *et al*., 1996; Alonso-Monge *et al*., 1999; Smith *et al*., 2004; Nicholls *et al*., 2009). There is some evidence that ubiquitination might also contribute to the regulation of some virulence factors in *C. albicans* (Leng *et al*., 2000; Roig and Gozalbo, 2003).

In *Saccharomyces cerevisiae* ubiquitin is encoded by a multigene family of natural gene fusions (Ozkaynak *et al*., 1987). *UBI1*, *UBI2* and *UBI3* encode hybrid proteins in which ubiquitin is fused to unrelated amino acid sequences (Ozkaynak *et al*., 1987). The fourth ubiquitin gene, *UBI4*, encodes a polyubiquitin comprising five consecutive head-to-tail ubiquitin repeats (Ozkaynak *et al*., 1987). After translation of this polyubiquitin polypeptide it is rapidly cleaved to generate ubiquitin monomers. *S. cerevisiae UBI4* is highly expressed under stress conditions such as elevated temperatures and starvation. Also, the inactivation of *UBI4* results in reduced resistance to starvation, growth at higher temperatures and sensitivity to amino acid analogues (Finley *et al*., 1987; Tanaka *et al*., 1988; Fraser *et al*., 1991). The global roles of ubiquitination in *C. albicans* have not been examined.

In *C. albicans*, polyubiquitin is encoded by the *UBI4* gene, which has three tandem repeats in a consecutive head-to-tail arrangement (Sepulveda *et al*., 1996; Roig *et al*., 2000). Downregulation of the *UBI4* gene in *C. albicans* induces the growth of hyphae and pseudohyphae (Roig and Gozalbo, 2003). This was consistent with a previous report indicating that the ubiquitination via the E2 enzyme Rad6 inhibits hyphal development in *C. albicans* (Leng *et al*., 2000). Downregulation of *UBI4* has also been reported to cause mild temperature sensitivity in stationary cells grown in glycerol, but not during growth on glucose (Roig and Gozalbo, 2003), suggesting that polyubiquitin might contribute to stress responses in *C. albicans*. It has been suggested that the *UBI4* gene is essential for viability in *C. albicans* (Roig and Gozalbo, 2003). These authors have also reported the existence of a second ubiquitin-encoding gene in *C. albicans* (Roig *et al*., 2000). *UBI3* encodes a protein fusion between a single unit of ubiquitin and ribosomal protein S34. blast searches of the *C. albicans* genome reveal no other ubiquitin-encoding sequences (Skrzypek *et al*., 2010).

Our aim in this study was to better understand the roles of the polyubiquitin gene in *C. albicans*. To achieve this we constructed independent null and conditional *C. albicans ubi4* mutants, indicating that the *UBI4* gene is not essential, as previously thought. *C. albicans ubi4/ubi4* cells were sensitive to a number of different physiologically relevant stresses. They also displayed defective metabolic adaptation, significant morphological abnormalities and unusual nuclear segregation in filamentous cells. Using a proteomic approach we identified 19 ubiquitinated *C. albicans* proteins with roles in growth, stress responses and metabolic adaptation, a finding that was entirely consistent with the phenotypes of *ubi4/ubi4* cells. Finally, we report that compromising Ubi4 function attenuates virulence in a murine model of systemic candidiasis. Clearly, ubiquitination plays important regulatory roles in key cellular processes that contribute to the pathogenicity of *C. albicans*.

## Results

### UBI4 is not essential for viability in *C. albicans*

**Fig. 1:**
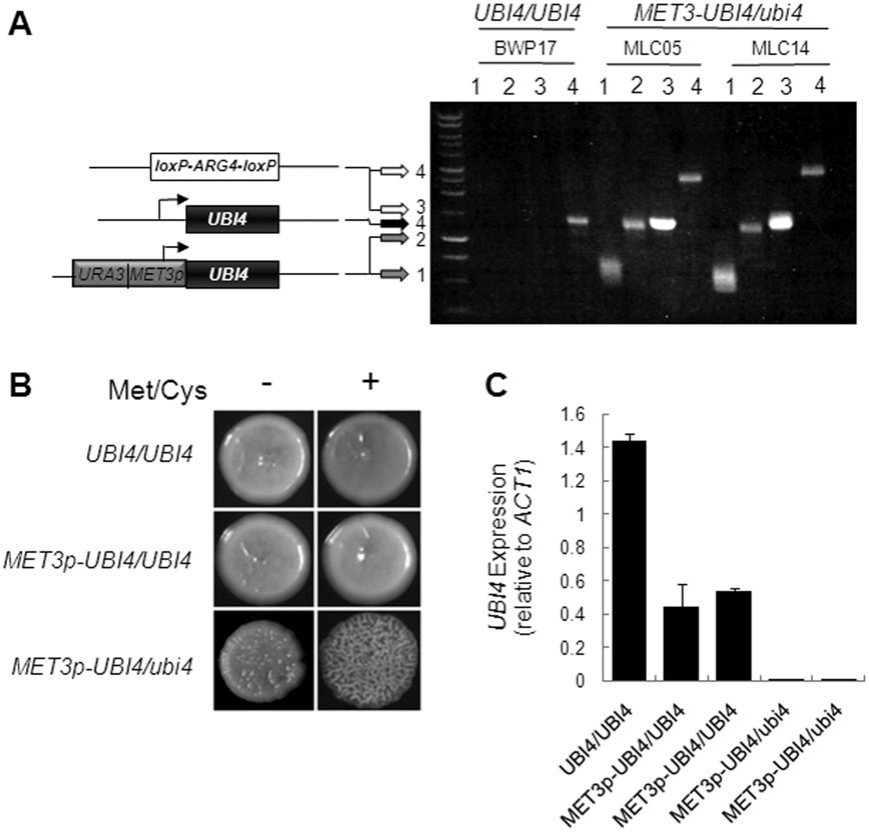
Downregulation of polyubiquitin expression in conditional *C. albicans MET3p-UBI4/ubi4* mutants does not prevent growth.A. Construction of the methionine-conditional *C. albicans* mutants MLC05 and MLC14 from the parental strain BWP17 (Table 1). One *UBI4* allele was disrupted by insertional inactivation using the *loxP-ARG4-loxP* cassette, and the other allele was placed under the control of the *MET3* promoter. Cartoons represent these alleles, and the arrows indicate the lengths of the corresponding diagnostic PCR products on the agarose gel: PCR reactions 1: primers MET3p-F and UBI4d-R (*Supporting information*– Primers); PCR reactions 2: primers MET3p-F and UBI4d2-R; PCR reactions 3: primers UBI4d-F and LALd-R; PCR reactions 4: primers UBI4d-F and UBI4d2-R.B. Growth of conditional *C. albicans MET3p-UBI4/ubi4* mutants in the presence of methionine and cysteine on plates. YPD plates contained (+) or lacked (−) 2.5 mM methionine (Met) and cysteine (Cys): *UBI4/UBI4* (BWP17); *MET3p-UBI4/UBI4* (MLC03); *MET3p-UBI4/ubi4* (MLC05) (Table 1).C. qRT-PCR quantification of *UBI4* mRNA levels, measured relative to the internal *ACT1* mRNA control, in cells grown with 2.5 mM Met/Cys for 4 h. Independent *MET3p-UBI4/UBI4* (MLC03, MLC12) and *MET3p-UBI4/ubi4* strains (MLC05, MLC14) were analysed.

The polyubiquitin gene, *UBI4*, is not essential in *S. cerevisiae*. However, previous attempts to create a homozygous *ubi4/ubi4* null mutation in *C. albicans* had failed (Roig and Gozalbo, 2003). For this reason we first created a methionine conditional *ubi4* mutant using the methionine/cysteine repressible *MET3* promoter (Care *et al*., 1999; Roig and Gozalbo, 2003). *C. albicans* is constitutively diploid. Hence, the first *UBI4* allele was placed under the control of the *MET3* promoter, and the second *UBI4* allele was deleted to create the independent conditional *C. albicans MET3p-UBI4/ubi4* mutants, MLC03 and MLC12 (Fig. 1A, Table 1).

**Table 1:** *Candida albicans* strains.

Strain	Genotype	Source
BWP17	*ura3Δ::λimm434/ura3Δ::λimm434 his1::hisG/his1::hisG arg4Δ::hisG/arg4::hisG*	Wilson *et al*. (1999)
THE1	*ade2::hisG/ade2::hisG, ura3::λ imm434/ura3::λ imm434, ENO1/eno1::ENO1-tetR-ScHAP4AD-3XHA-ADE2*	Nakayama *et al*. (2000)
MLC03	*ura3Δ::λimm434/ura3Δ::λimm434 his1::hisG/his1::hisG arg4Δ::hisG/arg4::hisG, URA3-MET3p-UBI4/UBI4*	This study
MLC05	*ura3Δ::λimm434/ura3Δ::λimm434 his1::hisG/his1::hisG arg4Δ::hisG/arg4::hisG, URA3-MET3p-UBI4/ubi4::loxP-ARG4-loxP*	This study
MLC12	*ura3Δ::λimm434/ura3Δ::λimm434 his1::hisG/his1::hisG arg4Δ::hisG/arg4::hisG, URA3-MET3p-UBI4/UBI4*	This study
MLC14	*ura3Δ::λimm434/ura3Δ::λimm434 his1::hisG/his1::hisG arg4Δ::hisG/arg4::hisG, URA3-MET3p-UBI4/ubi4::loxP-ARG4-loxP*	This study
MLC35	*ura3Δ::λimm434/ura3Δ::λimm434 his1::hisG/his1::hisG arg4Δ::hisG/arg4::hisG, UBI4/ubi4::loxP-URA3-loxP*	This study
MLC36	*ura3Δ::λimm434/ura3Δ::λimm434 his1::hisG/his1::hisG arg4Δ::hisG/arg4::hisG, UBI4/ubi4::loxP-URA3-loxP*	This study
MLC40	*ura3Δ::λimm434/ura3Δ::λimm434 his1::hisG/his1::hisG arg4Δ::hisG/arg4::hisG, ubi4::loxP-URA3-loxP/ubi4::loxP-ARG4-loxP*	This study
MLC41	*ura3Δ::λimm434/ura3Δ::λimm434 his1::hisG/his1::hisG arg4Δ::hisG/arg4::hisG, ubi4::loxP-URA3-loxP/ubi4::loxP-ARG4-loxP*	This study
MLC42	*ura3Δ::λimm434/ura3Δ::λimm434 his1::hisG/his1::hisG arg4Δ::hisG/arg4::hisG, ubi4::loxP-URA3-loxP/ubi4::loxP-ARG4-loxP*	This study
MLC60	*ura3Δ::λimm434/ura3Δ::λimm434 his1::hisG/his1::hisG arg4Δ::hisG/arg4::hisG, ubi4::loxP-URA3-loxP/ubi4::loxP-ARG4-loxP, RPS1::CIp30 (URA3, HIS1, ARG4)*	This study
MLC61	*ura3Δ::λimm434/ura3Δ::λimm434 his1::hisG/his1::hisG arg4Δ::hisG/arg4::hisG, ubi4::loxP-URA3-loxP/ubi4::loxP-ARG4-loxP, RPS1::CIp30-UBI4 (URA3, HIS1, ARG4)*	This study
MLC125	*ura3Δ::λimm434/ura3Δ::λimm434 his1::hisG/his1::hisG arg4Δ::hisG/arg4::hisG, RPS1::CIp30 (URA3, HIS1, ARG4)*	This study

To test the essentiality of *UBI4* in *C. albicans*, the wild-type parent (*UBI4/UBI4*), the heterozygous strain (*MET3p-UBI4/UBI4*) and the conditional mutants (*MET3p-UBI4/ubi4*) were plated on YPD medium containing or lacking 2.5 mM methionine and cysteine. As expected, all strains exhibited normal growth in the absence of methionine and cysteine (Fig. 1B). Also, the control strains grew normally in the presence of methionine and cysteine. Unexpectedly, *MET3p-UBI4/ubi4* cells also grew in the presence of methionine and cysteine, albeit displaying a wrinkly colonial phenotype (Fig. 1B), suggesting that *UBI4* is not essential for the growth of *C. albicans*. The strains were also examined in liquid culture, where they continued to grow in the presence of methionine and cysteine even after overnight incubation.

It was possible that the continued growth of *MET3p-UBI4/ubi4* cells in the presence of methionine and cysteine was due to ineffective repression of the *MET3p-UBI4* allele in our hands. To test this, we measured *UBI4* mRNA levels by qRT-PCR relative to the internal *ACT1* mRNA control (Fig. 1C). qRT-PCR was performed on RNA extracted from wild-type cells (BWP17: *UBI4/UBI4*) (Table 1), the conditional heterozygote (MLC03 and MLC12: *MET3p-UBI4/UBI4*) and the conditional mutants (MLC05 and MLC14: *MET3p-UBI4/ubi4*) grown for 4 h either in the absence or presence of 2.5 mM methionine and cysteine. As expected, following repression by methionine and cysteine, *UBI4* mRNA levels remained high in control cells, but were reduced to negligible levels in the conditional *MET3p-UBI4/ubi4* mutants (Fig. 1C).

**Fig. 2:**
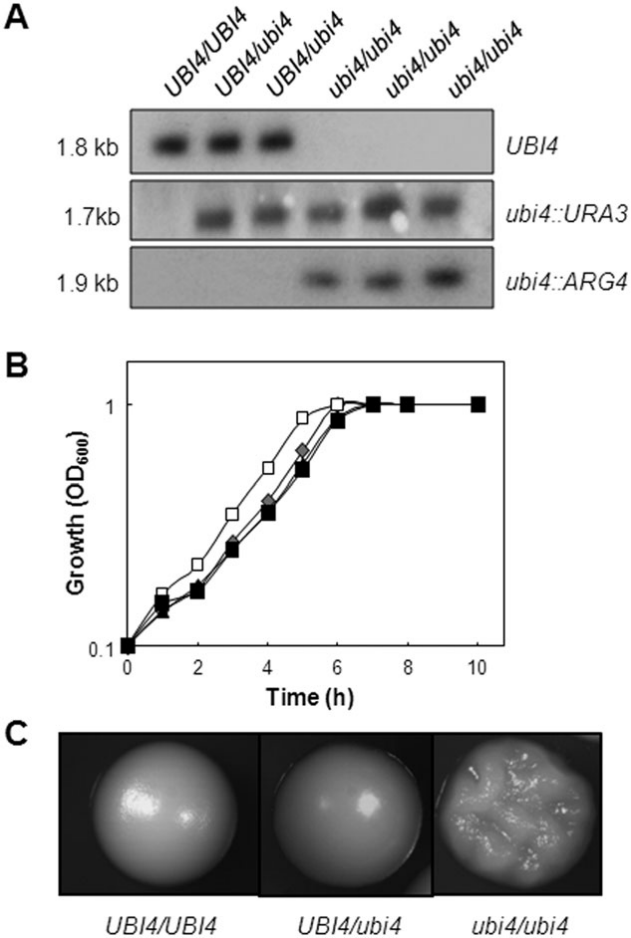
Polyubiquitin is not essential in *C. albicans*.A. The genotype of *C. albicans ubi4/ubi4* mutants was confirmed by Southern blotting of HindIII digested *C. albicans* genomic DNA with PCR-amplified probes against the *UBI4*, *ARG4* and *URA3* open reading frames: *UBI4/UBI4* (BWP17); *UBI4/ubi4* (MLC35 and MLC36); *ubi4/ubi4* (MLC40, MLC41, MLC42) (Table 1).B. Growth of *C. albicans ubi4/ubi4* mutants in liquid YPD at 30°C: *UBI4/UBI4*, grey diamonds (BWP17); *UBI4/ubi4*, open squares (MLC35); *ubi4/ubi4*, closed triangles and closed squares (MLC40, MLC41).C. Colonial growth of *C. albicans ubi4* mutants: *UBI4/UBI4* (BWP17); *UBI4/ubi4* (MLC35); *ubi4/ubi4 (*MLC40). Images were taken with the same magnification.

Although the *MET3p-UBI4* allele was effectively repressed by methionine and cysteine in the conditional mutant, some residual *UBI4* expression remained (Fig. 1C). Hence formally it remained possible that this residual *UBI4* expression was sufficient to support the growth of *C. albicans* (Fig. 1B). Therefore, we created independent *C. albicans ubi4/ubi4* null mutants (*ubi4::URA3/ubi4::ARG4*: MLC40, MLC41, MLC42: Table 1), confirming their genotypes both by Southern blotting (Fig. 2A) and diagnostic PCR. These *ubi4/ubi4* cells grew relatively normally on plates and in liquid media (Fig. 2B and C), thereby confirming that *UBI4* is not an essential gene in *C. albicans*.

### Polyubiquitin inactivation or depletion affects *C. albicans* cell morphology

**Fig. 3:**
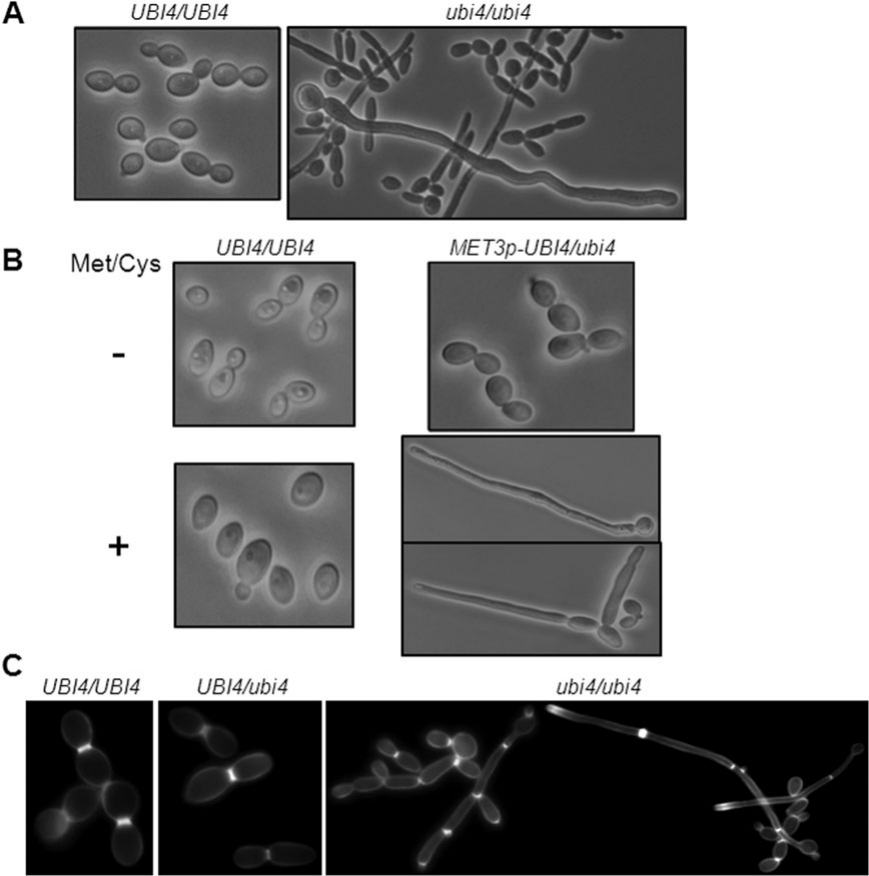
Inactivation or downregulation of polyubiquitin affects *C. albicans* cell morphology.A. Light microscopy of *C. albicans ubi4/ubi4* cells grown in YPD at 30°C reveals morphological abnormalities: *UBI4/UBI4* (BWP17); *ubi4/ubi4* (MLC40) (Table 1).B. Downregulation of *UBI4* in *C. albicans MET3p-UBI4/ubi4* cells with 2.5 mM methionine and cysteine (Met/Cys) essentially phenocopies the *ubi4/ubi4* null mutants: *UBI4/UBI4* (BWP17); *MET3p-UBI4/ubi4* (MLC05).C. Calcofluor White staining of *C. albicans ubi4/ubi4* cells reveals true hypha formation in some cells: *UBI4/UBI4* (BWP17); *UBI4/ubi4* (MLC35); *ubi4/ubi4* (MLC40).Images were taken at the same magnification and with the same exposures.

Roig and Gozalbo (2003) demonstrated that downregulation of *UBI4* using the methionine and cysteine-conditional *MET3* promoter affects colony morphology in *C. albicans*. They also suggested that Ubi4 depletion promotes hyphal and pseudohyphal growth in liquid culture although, as they pointed out, they were unable to draw a clear conclusion because the morphology of their control strains was also affected by methionine and cysteine addition (Roig and Gozalbo, 2003). For this reason we re-examined the impact of polyubiquitin depletion upon *C. albicans* morphology. The *C. albicans ubi4/ubi4* mutants formed wrinkly, rough, asymmetrical colonies compared with the smooth, waxy appearance of control colonies on YPD plates at 30°C (Fig. 2C). Furthermore, *ubi4/ubi4* cells also showed abnormal morphological phenotypes in liquid culture. Exponentially growing wild-type cells showed the expected budding yeast morphology in YPD at 30°C, whereas *ubi4/ubi4* cells formed a mixture of yeast, pseudohyphal and hyphal cells (Fig. 3A). Repression of the *MET3p-UBI4* allele in the conditional *MET3p-UBI4/ubi4* cells essentially phenocopied this behaviour of the *ubi4/ubi4* null mutant (Fig. 3B). Furthermore, the addition of methionine and cysteine had no effect upon the morphology of wild-type cells under our experimental conditions. These observations reinforced the suggestion of Roig and Gozalbo (2003) that the downregulation of *UBI4* promotes filamentous growth as well as our previous finding that ubiquitination is involved in morphogenetic regulation (Leng *et al*., 2000).

To examine septum formation in *C. albicans ubi4/ubi4* cells, we stained cells with Calcofluor White (Fig. 3C). As expected, wild-type budding cells displayed normal chitin distributions at septal junctions. Some filamentous *ubi4/ubi4* cells displayed septal plates within the germ tube, indicative of true hyphae (Sudbery, 2001). Other filamentous *ubi4/ubi4* cells were confirmed as pseudohyphae based on the location of their septal junctions at mother–daughter junctions (Fig. 3C). Similar morphological phenotypes were observed in the conditional *MET3p-UBI4/ubi4* mutant after growth in the presence of 2.5 mM methionine and cysteine. These data confirmed that the inactivation or depletion of polyubiquitin promotes mixed cell morphologies in *C. albicans*.

### Polyubiquitin inactivation or depletion affects nuclear segregation in *C. albicans*

**Fig. 4:**
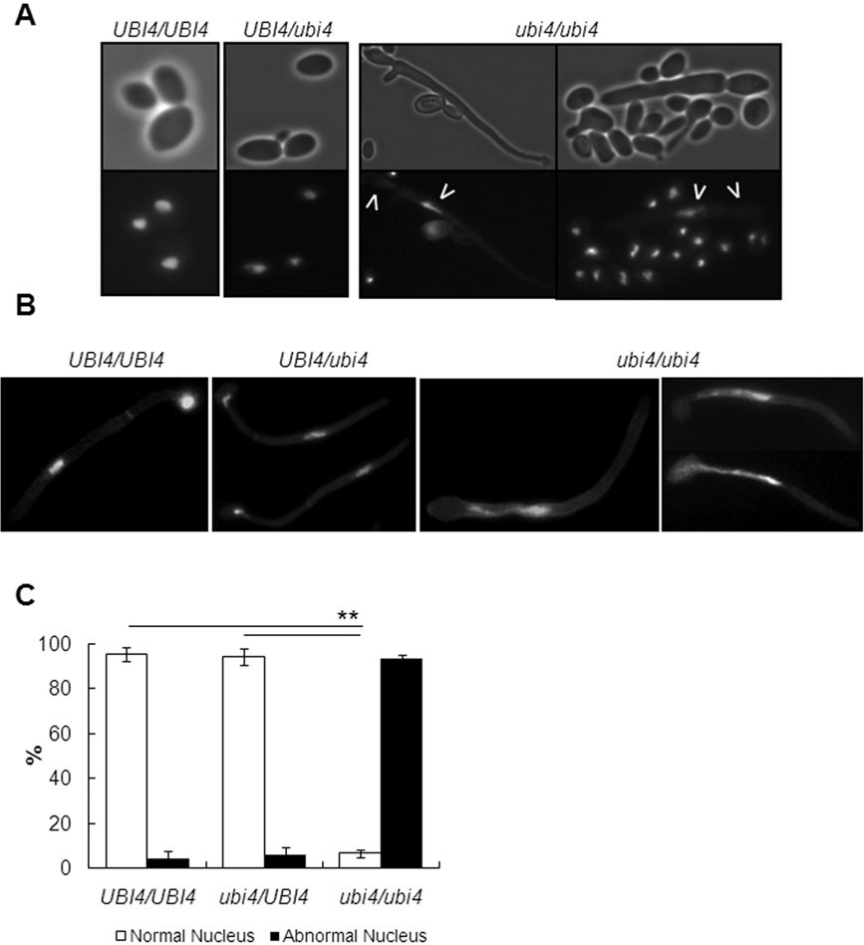
Inactivation of polyubiquitin affects *C. albicans* nuclear segregation.A. DAPI staining of *C. albicans ubi4/ubi4* cells grown in YPD at 30°C reveals defects in mitosis and aberrant nuclear segregation in some cells: *UBI4/UBI4* (BWP17); *UBI4/ubi4* (MLC35); *ubi4/ubi4* (MLC40).B. DAPI and Calcofluor White staining of *ubi4/ubi4* cells under hypha-inducing conditions highlights the extent to which nuclear segregation is incomplete: *UBI4/UBI4* (BWP17); *UBI4/ubi4* (MLC35); *ubi4/ubi4* (MLC40).C. Percentage of cells with aberrant nuclear segregation: *UBI4/UBI4* (BWP17); *UBI4/ubi4* (MLC35); *ubi4/ubi4* (MLC40). **P < 0.01.

Ubiquitination has been implicated in cell cycle regulation, for example through the degradation of cyclins (Glotzer *et al*., 1991). For this reason we examined the distribution of nuclei in *C. albicans ubi4/ubi4* cells by DAPI staining. Nuclear segregation appeared normal in *ubi4/ubi4* cells that grew with a yeast-like, budding morphology (Fig. 4A). However, defective nuclear segregation was apparent in those *ubi4/ubi4* cells displaying an aberrant filamentous morphology. In 91% of these filamentous cells, the ovoid mother cell lacked a nucleus, while the filamentous daughter cell contained an elongated nucleus. (Examples of this are illustrated in Fig. 4A.) Therefore, the altered morphology of some *ubi4/ubi4* cells correlated closely with defects in nuclear segregation. Similar observations were made following polyubiquitin depletion in the conditional *MET3p-UBI4/ubi4* mutant. We conclude that polyubiquitin contributes to the normal execution of the *C. albicans* cell cycle.

All of the above experiments were performed under conditions that normally promote growth of *C. albicans* in the yeast form. Do these observations hold under hypha-inducing conditions? To test this *C. albicans ubi4/ubi4* cells were compared with wild-type (*UBI4/UBI4*) and heterozygous control cells (*UBI4/ubi4*) after 2 h of growth at 37°C in YPD containing 10% serum. As expected, wild-type (*UBI4/UBI4*) and heterozygous (*ubi4/UBI4*) cells contained a single nucleus in the mother cell, and a second nucleus in the germ tube (Fig. 4B). In contrast, we observed that the nucleus was significantly extended from the mother cell into the germ tube in most cells lacking Ubi4 (*ubi4/ubi4*) (Fig. 4B and C). While only a small proportion of wild-type and heterozygous cells displayed an extended nucleus lying between ovoid mother cell and the daughter hyphal compartment (5% and 7% respectively), most mutant *ubi4/ubi4* cells (> 90%) displayed aberrant nuclear segregation (Fig. 4C). These data reinforce our conclusion that polyubiquitin contributes to the normal execution of the *C. albicans* cell cycle, particularly in filamentous growth.

### Polyubiquitin is required for stress adaptation in *C. albicans*

In *S. cerevisiae*, ubiquitination is regulated by a number of different stresses, and *UBI4* expression is increased in response to elevated temperatures and starvation. Furthermore, inactivating *ScUBI4* causes sensitivity to starvation, high temperatures and amino acid analogues (Finley *et al*., 1987; Tanaka *et al*., 1988; Fraser *et al*., 1991). We reasoned therefore, that *C. albicans ubi4/ubi4* cells might display a range of stress sensitivities. To test this, we compared the growth of wild-type (BWP17) and mutant cells (MLC40) under a variety of stress conditions.

**Fig. 5:**
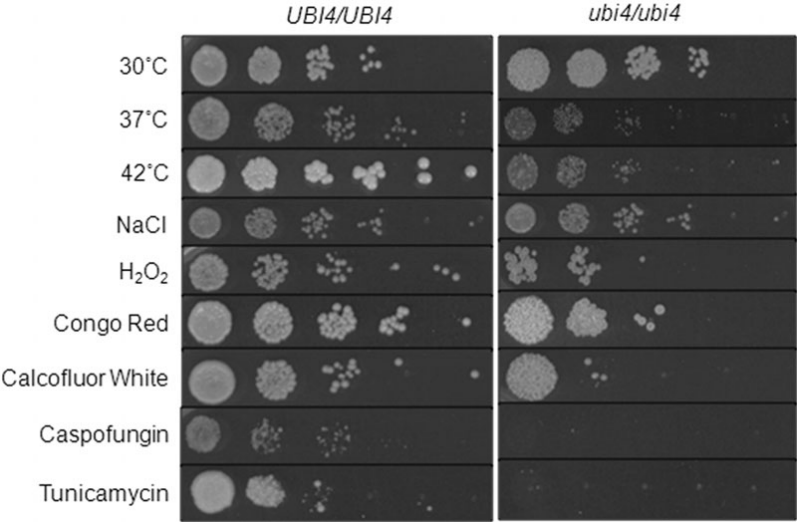
*Candida albicans* polyubiquitin mutants are sensitive to a range of stresses.A. Sensitivity of an *ubi4/ubi4* null mutant to stresses: *UBI4/UBI4* (BWP17); *ubi4/ubi4* (MLC40) (Table 1). Serial dilutions of exponentially growing cells were spotted onto YPD plates containing the appropriate stress at the following concentrations and examined after 48 h: H_2_O_2_ (5 mM), NaCl (1 M), Calcofluor White (20 µg ml^−1^), Congo Red (100 µg ml^−1^), tunicamycin (4.73 mM), caspofungin (0.064 µg ml^−1^) and thermal stress (37°C and 42°C).

*Candida albicans ubi4/ubi4* cells reproducibly displayed temperature sensitivity at 37°C and 42°C (Fig. 5). This observation was consistent with the suggestion that increased ubiquitination and proteasome-dependent degradation can alleviate the functions of heat shock proteins during stress (Friant *et al*., 2003). Although there was no obvious effect upon osmotic stress resistance, *UBI4* inactivation did render *C. albicans* cells more sensitive to peroxide (Fig. 5). This observation is significant in the context of infection as reactive oxygen species are important for the antimicrobial activity of host immune cells (Miller and Britigan, 1997). In addition, *C. albicans ubi4/ubi4* cells consistently displayed cell wall stress phenotypes. They grew more slowly in the presence of Congo Red and the anti-fungal drug, caspofungin, both of which target glucan biosynthesis. The *ubi4/ubi4* mutant was also sensitive to low concentrations of Calcofluor White, which affects chitin synthesis (Fig. 5). Furthermore, *ubi4/ubi4* cells were also sensitive to tunicamycin, which triggers the unfolded protein response (Wimalasena *et al*., 2008).

With the exception of peroxide and tunicamycin, these stress sensitivities were also examined in the conditional *C. albicans MET3p-UBI4/ubi4* mutant (*Supporting information*–*MET3p-UBI4* control). The impact of peroxide was not tested in the conditional *MET3p-UBI4/ubi4* mutant because of the confounding effects of the reducing agents methionine and cysteine. *C. albicans* cells were especially sensitive to tunicamycin in the presence of methionine and cysteine because both tunicamycin and reducing agents trigger the unfolded protein response in *C. albicans* (Wimalasena *et al*., 2008). Under repressing conditions the *C. albicans MET3p-UBI4/ubi4* mutant phenocopied *ubi4/ubi4* cells with respect to the other stress conditions we examined, displaying temperature and cell wall stress sensitivities (*Supporting information*–*MET3p-UBI4* control). In control experiments, the wild-type (BWP17) and *MET3p-UBI4/ubi4* cells (MLC05) displayed no significant differences under non-repressing conditions, i.e. in the absence of methionine and cysteine (*Supporting information*–*MET3p-UBI4* control). Collectively, our data indicate that polyubiquitin contributes to heat adaptation, the oxidative stress response and cell wall remodelling.

### Polyubiquitin is required for metabolic adaptation in *C. albicans*

Metabolic adaptation is central to the interactions that occur between *C. albicans* and its mammalian host during the infection process (Lorenz and Fink, 2001; Lorenz *et al*., 2004; Brown, 2005; Fradin *et al*., 2005; Thewes *et al*., 2007; Zakikhany *et al*., 2007). In other organisms, ubiquitination is not only important for the degradation of short-lived or damaged proteins (Bachmair *et al*., 1986; Jentsch *et al*., 1991), but also for the turnover of proteins during metabolic adaptation (Finley *et al*., 1987; Werner-Washburne *et al*., 1993; Muratani *et al*., 2005; Santt *et al*., 2008). Therefore, we reasoned that ubiquitination might play an important role during metabolic adaptation in *C. albicans*.

**Fig. 6:**
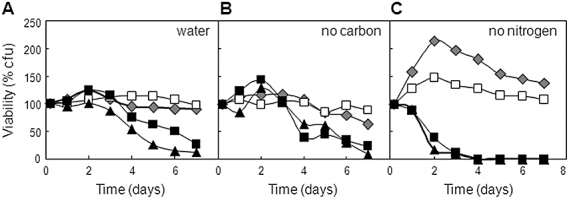
Inactivating polyubiquitin affects adaptation to nutrient starvation in *C. albicans*. Mid-exponential *C. albicans* cells were resuspended in minimal medium lacking a carbon and/or nitrogen source, and viability monitored thereafter (*Experimental procedures*): *UBI4/UBI4*, grey diamonds (BWP17); *UBI4/ubi4*, open squares (MLC35); *ubi4/ubi4*, closed triangles and closed squares (MLC40, MLC41) (Table 1).A. Carbon plus nitrogen starvation, where cells were incubated in water.B. Carbon starvation in YNB-C.C. Nitrogen starvation in YNB-N.

To test this, wild-type (BWP17), heterozygous *UBI4/ubi4* (MLC35) and homozygous *ubi4/ubi4* null cells (MLC40 and MLC41) were grown to mid-exponential phase in rich medium (YPD), and then transferred to starvation conditions. The viabilities of the wild-type and mutant cells were then compared at various times thereafter (Fig. 6). Wild-type and *UBI4/ubi4* cells retained their viability for a week after being starved in water, or after carbon deprivation (YNB-C: *Experimental procedures*). In contrast, the viabilities of two independent *ubi4/ubi4* null strains declined after these different types of starvation were imposed (Fig. 6A and B). An even more dramatic phenotype was observed when cells were starved for nitrogen in the presence of excess carbon (YNB-N: *Experimental procedures*). The *ubi4/ubi4* cells rapidly lost their viability under these conditions, whereas wild-type and *UBI4/ubi4* control cells not only remained viable, but displayed limited growth (Fig. 6C). This phenotype was not due to marker effects. Strains in which all auxotrophic markers were reinstated displayed similar starvation sensitivities to those shown in Fig. 6 (MLC60, MLC61, MLC125: Table 1). We conclude that, like the situation in *S. cerevisiae* (Finley *et al*., 1987; Werner-Washburne *et al*., 1993), polyubiquitin is clearly required for the survival of *C. albicans* under starvation conditions, and particularly when nitrogen is limiting but carbon is in excess. This is entirely consistent with a major role for ubiquitin-mediated protein degradation during the metabolic adaptation of this pathogen to starvation conditions.

### UBI4 is required for the ubiquitination of numerous proteins in *C. albicans*

In *S. cerevisiae*, ubiquitination influences numerous processes including metabolic adaptation, the cell cycle, protein transport, transcription and stress responses (Peng *et al*., 2003; Mayor *et al*., 2005; Tagwerker *et al*., 2006; Seyfried *et al*., 2008). However, little is known about ubiquitination targets in *C. albicans*, although ubiquitination probably affects many processes that underpin the virulence of this fungal pathogen. Based on our phenotypic analysis, we reasoned that a number of proteins must be targeted by *UBI4*, and therefore our next aim was to identify ubiquitinated proteins in *C. albicans*.

Epitope tagging of Ubi4 was not feasible because polyubiquitin is cleaved into single units for ubiquitination. Therefore, we used an anti-ubiquitin antibody to examine ubiquitinated protein targets in *C. albicans*. First, we examined ubiquitination in *C. albicans* cells under a range of experimental conditions: heat shock (30–42°C for 1 h), oxidative stress (50 mM H_2_O_2_ for 1 h), cell wall stress (SDS, Congo Red and Calcofluor White for 1 h), serum (10% fetal calf serum for 1 h) and control untreated cells. Protein extracts were then prepared from these cells and subjected to Western blotting with the anti-ubiquitin antibody.

**Fig. 7:**
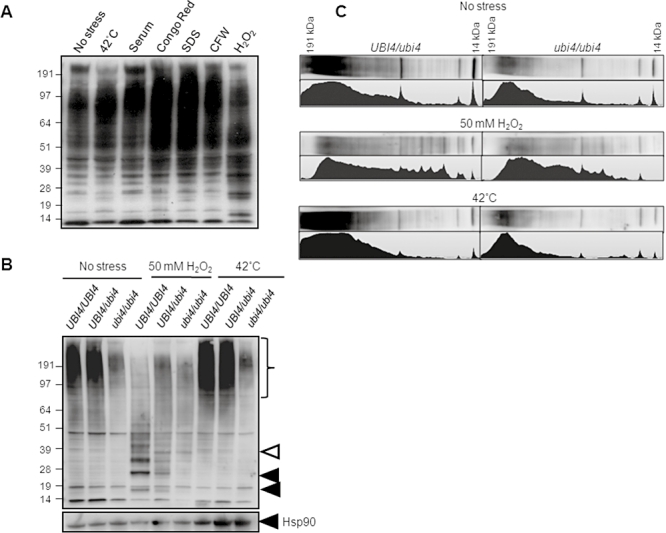
Many *C. albicans* proteins are ubiquitinated in a polyubiquitin-dependent fashion.A. Protein ubiquitination in wild-type *C. albicans* cells under different stress conditions. *C. albicans* THE1 was grown for 5 h and then exposed to the specified stress for 1 h (*Experimental procedures*). Protein extracts were prepared and analysed by Western blotting with an α-ubiquitin antibody.B. Impact of *UBI4* inactivation upon protein ubiquitination in *C. albicans*. *UBI4/UBI4* (THE1), *UBI4/ubi4* (MLC35) and *ubi4/ubi4* (MLC40) cells were grown for 5 h and then stressed for 1 h with heat or peroxide (*Experimental procedures*). Protein extracts were prepared and analysed by Western blotting. To control for loading, membranes were stripped and reprobed for Hsp90. Closed arrows highlight bands observed in wild-type cells that are absent following *UBI4* inactivation, and the open arrow indicates a band observed in heat stressed cells that is absent in the *ubi4/ubi4* mutant. The bracket indicates numerous ubiquitinated proteins, the levels of which are decreased in *ubi4/ubi4* cells.C. Quantification of protein levels in the heterozygous *UBI4/ubi4* (MLC35) and null *ubi4/ubi4* (MLC40) strains under the three conditions tested.

A large number of ubiquitinated proteins were observed in untreated *C. albicans* cells, and these patterns of ubiquitination changed following heat shock, or after exposure to cell wall stress or peroxide (Fig. 7A). For example, peroxide stress led to the appearance of a number of ubiquitinated proteins of between 20 and 50 kDa. Also, a new band of about 23 kDa was apparent following heat stress, as well as additional ubiquitinated proteins of masses between 60 and 100 kDa. Following exposure to cell wall stresses, heavy ubiquitination was observed on proteins of between 50 and 100 kDa. These different ubiquitination patterns were consistent with our working hypothesis that ubiquitination plays significant roles in *C. albicans* during stress adaptation.

We chose to examine ubiquitination following peroxide treatment and heat stress because *C. albicans* is exposed to oxidative stress during contact with host immune defences (Lorenz *et al*., 2004; Fradin *et al*., 2005; Enjalbert *et al*., 2007) and because ubiquitination has been shown to play a significant role in *S. cerevisiae* during the heat shock response (Friant *et al*., 2003). However, before doing so, we tested whether *UBI4* contributes significantly to ubiquitination under these experimental conditions. This is because in *C. albicans* ubiquitin is derived from Ubi3 as well as Ubi4. To do this we examined the patterns of ubiquitination in wild-type (*UBI4/UBI4*), heterozygous (*UBI4/ubi4*) and homozygous null cells (*ubi4/ubi4*). Protein extracts from untreated, heat-shocked or peroxide-treated cells were examined by Western blotting and the signals quantified (Fig. 7B and C). *UBI4* inactivation led to significant reductions in ubiquitination under all of the conditions examined, and in particular to the loss of some ubiquitinated proteins in the 20–50 kDa range following peroxide stress (Fig. 7C). This observation was strengthened by Ponceau S staining of the membranes, which confirmed that the protein loadings on the Western blots were reasonably even (*Supporting information*– Ponceau S Stain). We also reprobed the membranes for Hsp90 (Fig. 7B). Hsp90 levels were similar, except following heat shock, when Hsp90 levels increased as expected (Swoboda *et al*., 1995; Nicholls *et al*., 2009).

We conclude that a significant proportion of the ubiquitination observed during peroxide or heat stress is performed using ubiquitin derived from *UBI4*, and that residual ubiquitination under these conditions employs ubiquitin from *UBI3*. This conclusion is consistent with the fact that *UBI4* expression is upregulated in response to stress (Brown *et al*., 2000; Roig and Gozalbo, 2002).

### Identification of ubiquitinated proteins in *C. albicans*

**Fig. 8:**
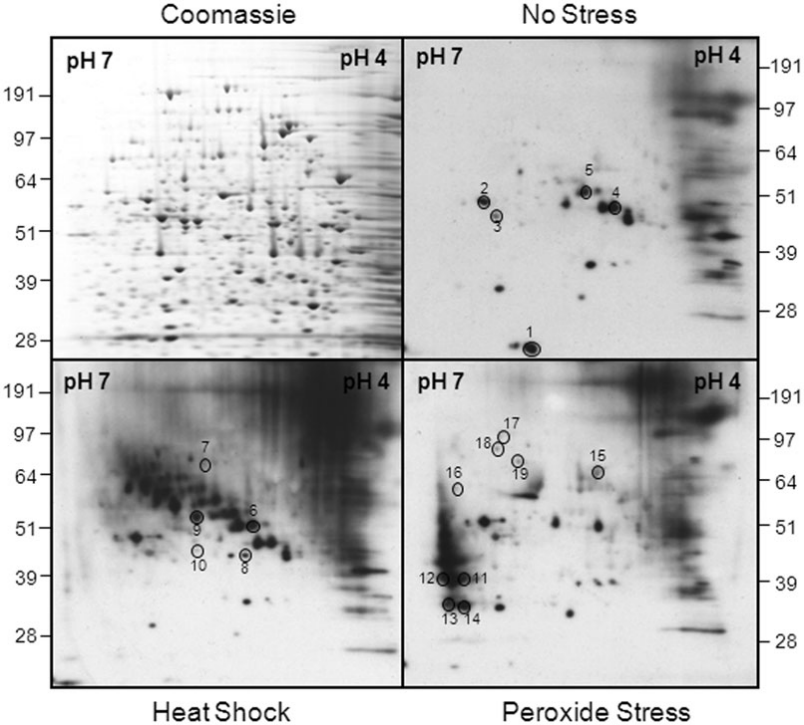
Identification of ubiquitinated proteins in *C. albicans* using a proteomic screen. *C. albicans* THE1 cells were grown for 5 h to mid-exponential phase and then exposed to stress for 1 h. Protein extracts were run on replicate 2-D gels, which were either stained with Coomassie blue or subjected to Western blotting with an α-ubiquitin antibody: Western blots of no stress control; peroxide-treated cells (50 mM H_2_O_2_); heat-shocked cells (30–42°C). Autoradiographs were aligned with the Coomassie-stained gels, spots chosen for analysis, and the corresponding proteins identified by tryptic digestion and LC-MS/MS (see text). The sample reference numbers for ubiquitination targets are shown (Table 2).

Having established that *UBI4* plays a significant role in ubiquitination during responses to heat shock and peroxide, we attempted to identify protein targets of ubiquitination under these conditions. Exponentially growing *C. albicans* cells were exposed to a 30–42°C heat shock or to 50 mM H_2_O_2_ for 1 h, while control cells remained untreated. Protein extracts from these cells were subjected to 2-D gel electrophoresis, and replicate 2-D gels were stained with Coomassie blue or subjected to Western blotting using the anti-ubiquitin antibody (Fig. 8). Ubiquitinated proteins were identified by aligning the Western blots with the Coomassie-stained gels, and only those spots that displayed reproducible regulation in two biologically independent experiments were examined further. These spots of interest were then cut from the Coomassie-stained gels and the corresponding proteins identified by tryptic digestion followed by tandem mass spectrometry (*Experimental procedures*).

**Table 2:** Identification of ubiquitinated proteins in *C. albicans*.

Sample ref	Accession number	Protein name	Molec. Wt. (Da)	pI	Function	Ubiquitinated in *S. cerevisiae*?	Ubiquitination detected in absence of stress?	Predicted ubiquitination site(s)
Constitutively ubiquitinated								
1	Ca2937	RPS21B	8 807	8.85	Ribosomal protein S21	ND	Yes	Medium confidence, position 5
2	Ca1691	PGK1	45 266	6.07	Phosphoglycerate kinase	Yes	Yes	Medium confidence, position 6; low confidence, position 296
3	Ca5180	FBA1	39 362	5.69	Fructose-bisphosphate aldolase	Yes	Yes	Medium confidence, position 72 and 192; low confidence, position 77
4	Ca2939	TIF1	44 742	5.22	Translation initiation factor	Yes	Yes	Low confidence, position 24
4	Ca0824	GPD2	41 174	5.21	Glycerol-3-phosphate dehydrogenase	Yes	Yes	Low confidence, position 274 and 318
Increased ubiquitination in response to heat								
5	Ca3874	ENO1	47 202	5.54	Enolase	Yes	Yes	High confidence, position 273; medium confidence, position 265; low confidence, position 432
6	Ca4389	RPT5	47 814	5.25	Regulatory particle triphosphatase	Yes	Yes	Medium confidence, position 55, 63, 163, 170, 284 and 290; low confidence, position 35 and 92
7	Ca3924	TKL1	73 841	5.48	Transketolase	Yes	No	Low confidence, position 351
8	Ca2895	PRN4	37 072	5.31	Putative pirin by homology	ND	No	High confidence, position 315; medium confidence, position 234; low confidence, position 240, 320 and 327
9	Ca5239	GND1a	57 159	6.14	6-Phosphogluconate dehydrogenase	Yes	No	Medium confidence, position 6; low confidence, position 151 and 388
9	Ca2011	UBI3	17 485	9.86	Functional homologue of *S. cerevisiae* RPS31	ND	No	Medium confidence, position 105
10	Ca0210	orf19.5525	37 783	5.53	Putative NADP(H) oxidoreductase	ND	No	Medium confidence, position 285; low confidence, position 268 and 344
Increased ubiquitination in response to H_2_O_2_								
11	Ca4261	PDX3	28 773	6.02	Pyridoxine (pyridoxamine) phosphate oxidase	Yes	No	Medium confidence, position 22 and 58
12	Ca2675	GSP1a	24 470	6.53	Small RAN G-protein	Yes	No	Medium confidence, position 193
12	Ca5932	UBI4	25 776	7.76	Ubiquitin precursor (polyubiquitin)	Yes	No	Low confidence, position 33, 48, 63, 139, 179 and 215
13	Ca1673	PST2	21 714	6.51	NADH : quinone oxidoreductase	Yes	No	None
13	Ca5037	PRE3	23 629	6.42	Beta-1 subunit of proteasome	ND	No	Low confidence, position 12
14	Ca4791	PRE1a	22 119	6.43	β4 subunit of the 20S proteasome	ND	No	Low confidence, position 19
14	Ca4589	ASC1a	23 619	6.3	40S ribosomal subunit protein	Yes	No	None
14	Ca5932	UBI4	25 776	7.76	Ubiquitin precursor (polyubiquitin)	Yes	No	Low confidence, position 33, 48, 63, 139, 179 and 215
Increased ubiquitination in response to heat and H_2_O_2_								
15	CA3534	SSB1	66 580	5.25	HSP70 family chaperone	Yes	No	High confidence, position 528 and 545; medium confidence, position 521, 530, 543 and 597; low confidence, position 191, 314 and 393
16	Ca1246	IMH3	40 055	6.67	Inosine monophosphate (IMP) dehydrogenase	ND	No	Medium confidence, position 7; low confidence, position 24, 175, 230 and 517
17	Ca3546	ACO1	84 625	5.96	Aconitase	ND	No	Medium confidence, position 25.
18	Ca2858	ACS2	74 215	5.73	Acetyl-CoA synthetase	Yes	No	Medium confidence, position 32 and 596; low confidence, position 17 and 583
19	Ca2470	SDH12	70 930	6.03	Flavoprotein subunit of succinate dehydrogenase	ND	No	Medium confidence, position 480; low confidence, position 417, 492 and 500

a Protein detected with ubiquitin sequences.

ND, not detected.

Table 2 lists those protein identifications that met several strict data quality criteria: a Mascot score of > 35 (*P* < 0.05), plus an Excess of Limit-Digested Peptides (EDLP) score of ≥ 1 (Stead *et al*., 2006). The quality metrics for these protein identifications are provided in the *Supporting information* (Ubiquitination Targets). For those *C. albicans* proteins that were identified using our proteomic approach, we looked for experimental evidence for ubiquitination of their *S. cerevisiae* orthologue. In addition, we screened for potential ubiquitination sites in the *C. albicans* protein sequence using the program UbPred (Radivojac *et al*., 2010) (Table 2).

This was not an exhaustive proteomic screen for ubiquitinated target proteins in *C. albicans*. For example, some ubiquitinated proteins in *C. albicans* might lie outside the pI range of our 2-D gels, or they might not be expressed under the conditions examined. Furthermore, some ubiquitin targets might be low abundance proteins that lie below the sensitivity of our analyses. Nevertheless, our aim in these experiments was to identify some proteins that are ubiquitinated in stressed or control cells, and some proteins whose ubiquitination increases either in response to heat shock or peroxide stress. Some of the gel spots we analysed contained more than one protein. In this case, we looked for evidence of ubiquitination in the *S. cerevisiae* orthologue, for potential ubiquitination sites in the *C. albicans* protein, and protein identifications with the most significant Mascot scores. In a small number of cases, we found that of two proteins identified, one is ubiquitinated in *S. cerevisiae* but contains no potential ubiquitination sites in the *C. albicans* sequence, whereas the other protein does contain a ubiquitination site but has not been shown to be ubiquitinated in *S. cerevisiae*. In these circumstances it was not possible to prioritize the likely ubiquitination target. Furthermore, it was conceivable that more than one protein in a particular 2-D spot was ubiquitinated. Therefore, under these circumstances both protein identifications were retained in Table 2. Nevertheless, 19 ubiquitination features were identified in our proteomic screen, the orthologues of many of which are ubiquitinated in *S. cerevisiae*. Furthermore, our identification of ubiquitin sequences with a number of these targets reinforced the validity of this screen (Table 2).

As mentioned, our proteomic approach revealed *C. albicans* proteins whose orthologues have been shown to be ubiquitinated in *S. cerevisiae* (Table 2). These included metabolic proteins (Eno1, Fba1, Gpd2, Pgk1, Pdx3, Tkl1, Acs2) a heat shock protein (Ssb1) and oxidative stress proteins (Gnd1, Pst2). Furthermore, proteins involved in the degradation of ubiquitinated substrates were also detected (Rpt5, Ubi4 and Ubi3), as well a translation factor (Tif1) and a cell cycle protein (Gsp1). Moreover, Pre3 and Pre1, proteins of the 20S proteosome involved in ubiquitin-dependent degradation were also detected. These proteins lend credence to our list of ubiquitination targets in *C. albicans*.

Some of the ubiquitination targets we identified appeared to be constitutively ubiquitinated in *C. albicans* based on the intensity of their signals on Western blots, whereas the ubiquitination of other targets appeared to increase in response to heat shock and/or oxidative stress (Fig. 8, Table 2). In many cases the functions of these ubiquitination targets appeared to correlate with their patterns of ubiquitination. For example, the ubiquitination of the heat shock protein (Ssb1) and oxidative stress proteins (Gnd1, Pst2) increased in response to stress, whereas other proteins, such as the metabolic enzymes Eno1, Pgk1 and Fba1, were constitutively ubiquitinated. In particular, a set of proteins of between 20 and 50 kDa were ubiquitinated in response to peroxide stress, and their ubiquitination was Ubi4-dependent (Fig. 7B). Our proteomic analyses identified four peroxide stress-induced features in this category (spots 8, 10–14: Fig. 8), including an oxidoreductase (Pst2) and two proteasomal subunits (Pre1, Pre3) (Table 2). Two of these spots contained ubiquitin sequences, thereby reinforcing their validity as ubiquitination targets. Obviously the aberrant ubiquitination of these targets in *ubi4/ubi4* cells could contribute to their peroxide sensitivity (Fig. 5). Therefore, the proteomic data further supported the view that ubiquitination contributes significantly to environmental adaptation in *C. albicans* and provides some mechanistic explanations for the phenotypes of the *ubi4/ubi4* mutants.

### Polyubiquitin contributes significantly to virulence in *C. albicans*

**Fig. 9:**
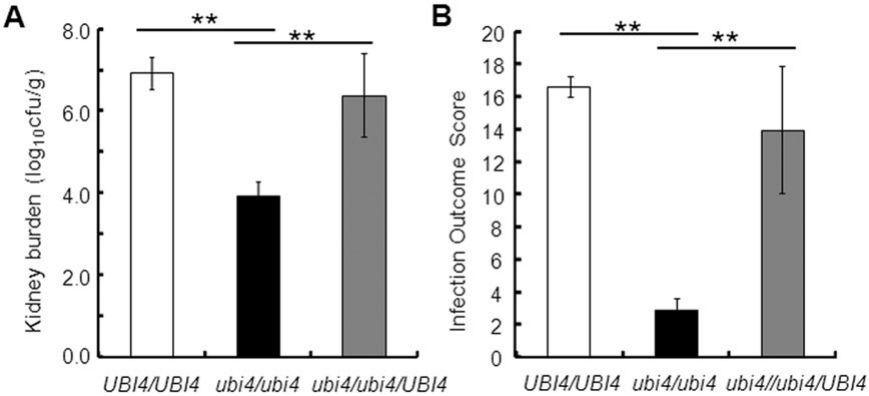
Inactivation of polyubiquitin significantly attenuates the virulence of *C. albicans* in a mouse model of systemic candidiasis.A. Kidney burdens measured at 72 h post infection (means and standard deviations for six animals): *UBI4/UBI4*, MLC125, white bar; *ubi4/ubi4*, MLC60, black bar; *ubi4/ubi4/UBI4*, MLC61, grey bar (Table 1).B. Infection outcome scores calculated after 3 days (means and standard deviations for six animals), where higher outcome scores reflect more severe infection.In both panels, **P < 0.01.

The above observations suggested that ubiquitination might contribute significantly to the physiological fitness of *C. albicans* cells *in vivo*, and hence, that Ubi4 might contribute to the virulence of this pathogen. To test this mice were infected with *C. albicans ubi4/ubi4* cells (MLC60), or with the control strains MLC125 (*UBI4/UBI4*) and MLC61 (*ubi4/ubi4/UBI4*). All of these strains were prototrophic, the markers having been restored by integration of the plasmid CIp30 (Dennison *et al*., 2005). Virulence was assayed by determining infection outcome scores, based upon weight change and kidney burdens at 3 days post infection (*Experimental procedures*; MacCallum *et al*., 2010). Statistical analyses revealed no significant difference between the wild-type (MLC125) and reintegrant control strains (MLC61) for any of the parameters tested (weight loss, kidney fungal burden and outcome score) (Fig. 9A). However, when mice were infected with the *ubi4/ubi4* null mutant (MLC60), they exhibited significantly lower kidney fungal burdens and outcome scores compared with the control strains; *P* < 0.01 (Fig. 9A and B). Taken together these data clearly indicate that *UBI4* inactivation attenuates the virulence of *C. albicans*.

## Discussion

In this study we combined molecular, cellular and proteomic approaches to examine the roles of ubiquitination, and polyubiquitin in particular, in the fungal pathogen *C. albicans*. A number of significant conclusions can be drawn from our datasets.

First we have shown that, despite the critical roles for ubiquitination in *C. albicans*, the polyubiquitin gene, *UBI4*, is not essential for viability (Fig. 2). The generation of a homozygous *C. albicans ubi4/ubi4* mutant proved difficult in the past (Roig and Gozalbo, 2003). However, independent *ubi4/ubi4* mutants were successfully generated in this study (Fig. 2), and their phenotypes were replicated by independent methionine-conditional *C. albicans MET3p-UBI4/ubi4* mutants (Fig. 3 and *Supporting information*–*MET3p-UBI4* control). The growth of *ubi4/ubi4* cells is almost certainly supported by the expression of residual amounts of ubiquitin, even in the absence of the Ubi4 polyubiquitin protein (Fig. 7B and C). We presume that this residual ubiquitin is derived from the only other ubiquitin-encoding locus in the *C. albicans* genome, *UBI3*.

The second conclusion is that ubiquitination appears to contribute to cell cycle progression and morphogenesis in *C. albicans*. This conclusion is based on our observation that a subset of *ubi4/ubi4* cells displays both aberrant nuclear segregation patterns and abnormal filamentous morphologies (Figs 3 and 4). However, if *UBI4* plays a role in *C. albicans* cell cycle regulation, why did *ubi4/ubi4* cells display population heterogeneity (Fig. 3)? Presumably the residual ubiquitin encoded by *UBI3* is sufficient to drive cell cycle regulation in most *ubi4/ubi4* cells. However, these residual ubiquitin levels might fall below critical levels in a subset of *ubi4/ubi4* cells, thereby leading to the observed defects in morphology and nuclear segregation. Therefore, stochasticity in residual ubiquitin levels might account for the observed population heterogeneity of *ubi4/ubi4* cells (Fig. 3).

Why might the defects in morphology and nuclear segregation correlate so strongly? Our data are consistent with the idea that ubiquitin depletion preferentially inhibits nuclear division in filamentous, rather than budding cells. According to this working model, once ubiquitin levels fall below a critical level, this probably triggers hyphal development in this *C. albicans* cell (Leng *et al*., 2000). The migration of the maternal nucleus into this new germ tube might proceed normally (Sudbery, 2001), but because ubiquitin levels are abnormally low in this cell, nuclear division is inhibited, thereby preventing the subsequent migration of the daughter nuclei into the mother and daughter compartments. This working model can account for our observations.

How might ubiquitination influence morphology and cell cycle-related events in *C. albicans*? Gsp1 is a ubiquitinated protein in *C. albicans* (Table 2). This Ran GTPase is also ubiquitinated in *S. cerevisiae*, where it is involved in the maintenance of nuclear and chromosome organization (Tagwerker *et al*., 2006). Overexpression of Gsp1 causes aberrant cell cycle progression in *S. cerevisiae* (Stevenson *et al*., 2001), and therefore aberrant ubiquitination of Gsp1 in *C. albicans* might account for the defective cell cycle progression in some *ubi4/ubi4* cells (Fig. 4A). Genes encoding other ubiquitinated proteins in *C. albicans* have links to morphogenesis (Table 2). *PDX3* is regulated during hyphal development (Nantel *et al*., 2002) and *GPD2* is regulated by Ssn6, a negative regulator of morphogenesis (Hwang *et al*., 2003; Garcia-Sanchez *et al*., 2005). Interestingly, Ssn6 also regulates *UBI4* expression (Garcia-Sanchez *et al*., 2005). While these links between ubiquitination and morphogenesis may appear relatively tenuous, they illustrate the important point that ubiquitination might influence *C. albicans* cell morphology indirectly via a number of cellular processes including cell cycle progression, defects in which are known to affect filamentation in *C. albicans* (Andaluz *et al*., 2001; Shi *et al*., 2007).

However, more direct links exist between ubiquitination and morphogenesis in *C. albicans*. Rad6, which is an E2 ubiquitin-conjugating enzyme that targets proteins for degradation via the proteasome (Madura and Varshavsky, 1994), is a negative regulator of hyphal development in *C. albicans* (Leng *et al*., 2000). The prevailing view is that Rad6 targets a component on the Ras-cAMP-Efg1 signalling pathway for ubiquitination-mediated protein degradation. Furthermore the inactivation of Doa1, which plays a role in the ubiquitin pathway (Hochstrasser *et al*., 1995; Johnson *et al*., 1995), promotes filamentous growth in *C. albicans* (Kunze *et al*., 2007). Therefore, ubiquitination probably influences *C. albicans* morphogenesis in several ways: directly through regulation of morphogenetic signalling events, and indirectly through modulation of cell cycle progression. Whatever the mechanisms, the observation that inactivation or downregulation of *UBI4* affects colonial and cellular morphology in *C. albicans* is consistent with the work of Roig and Gozalbo (2003). It is also consistent with previous work from our laboratory implicating ubiquitination in the regulation of the yeast–hypha transition in *C. albicans* (Leng *et al*., 2000).

Our third main conclusion is that polyubiquitin is required for stress adaptation in *C. albicans*. *C. albicans ubi4* mutants showed susceptibility to a wide range of stresses (Fig. 5). These defects were specific because, while *C. albicans ubi4/ubi4* cells were sensitive to some conditions (such as elevated temperatures, cell wall and oxidative stresses), they were resistant to others (such as osmotic stress). The temperature-sensitive phenotype was consistent with the observation that *UBI4* expression is upregulated in response to thermal upshifts (Roig and Gozalbo, 2002). The stress sensitivities of *ubi4/ubi4* cells were also consistent with the findings that ubiquitination is stimulated by stresses in *C. albicans* (Fig. 7) and in *S. cerevisiae* (Cheng *et al*., 1994). Interestingly, although ubiquitination has been extensively studied in *S. cerevisiae*, there are no reports of ubiquitin-related osmotic stress phenotypes. This is entirely consistent with our observation that *C. albicans ubi4/ubi4* cells are as resistant to osmotic stresses as wild-type cells (Fig. 5). Furthermore, proteomic screens in *S. cerevisiae* have shown that numerous heat shock proteins are ubiquitinated in this yeast. These include Hsc82, Hsp104 Ssa1, Ssa2, Ssa3, Ssa4, Ssb1, Ssb2 and Kar2 (Mayor *et al*., 2005; Tagwerker *et al*., 2006). Once again, this was consistent with our identification of Ssb1 as a ubiquitination target in *C. albicans* (Table 2), and other Hsp70 family members are likely to ubiquitinated in this pathogen as Hsp70, Ssa2 and Kar2, for example, all carry potential ubiquitination sites. In addition, we identified the oxidative stress response proteins Gnd1 and Pst2 as ubiquitination targets in *C. albicans*. The ubiquitination of Pst2 was induced by peroxide stress (Table 2). Furthermore, the peroxide-induced ubiquitination of several proteins was significantly attenuated in *ubi4/ubi4* cells (Fig. 7B). Clearly, aberrant ubiquitination of Gnd1 and Pst2 could account, at least in part, for the stress sensitivities of *C. albicans ubi4/ubi4* cells (Fig. 5).

The fourth conclusion is that polyubiquitin contributes significantly to metabolic adaptation in *C. albicans*. Our proteomic data and phenotypic analyses, combined with observations in other systems, strongly support this conclusion. Numerous metabolic enzymes were identified as ubiquitination targets in *C. albicans*, most of which are involved in carbon metabolism (Table 2). Enzymes on the following pathways were identified in our screen: glycolysis/gluconeogenesis (Eno1, Fba1, Pgk1); pentose phosphate pathway (Tkl1); fatty acid metabolism (Pdx3); acetate utilisation (Asc2); and glycerol synthesis (Gpd2). It should be noted that the loss of polyubiquitin affected the ability of *C. albicans* to survive under all of the starvation conditions examined (Fig. 6). However, the strongest metabolic phenotype was not observed when *C. albicans* cells were starved for carbon, but when cells were starved for nitrogen in the presence of excess carbon (Fig. 6C). Under these conditions *ubi4/ubi4* cells rapidly lost their viability while control cells were able to support some growth.

The *S. cerevisiae* proteome undergoes considerable remodelling in response to nutrient limitation (Werner-Washburne *et al*., 1993), with proteins involved in protein turnover and nitrogen source scavenging being upregulated following nitrogen starvation (Kolkman *et al*., 2006). Furthermore, nitrogen recycling enzymes such as glutamine synthetase are ubiquitinated in *S. cerevisiae* (Peng *et al*., 2003; Tagwerker *et al*., 2006). We did not identify Gln1 in our screen. However, our bioinformatic analyses revealed that it contains four possible ubiquitination sites, indicating that it may still be a target in *C. albicans*. Nevertheless, we suggest that the ubiquitinated metabolic enzymes that we did identify reflect the recycling of available nitrogen in *C. albicans*, rather than the reprogramming of metabolism *per se*. Metabolic reprogramming undoubtedly takes place in *C. albicans* cells following starvation. This view is supported by the transcriptomic studies of Lorenz and co-workers (Lorenz *et al*., 2004), and by the observation that *UBI4* is upregulated during starvation (Roig and Gozalbo, 2002). Nevertheless, the ubiquitination of glycolytic enzymes, for example, might reflect the fact that these are abundant proteins that are present in excess in *C. albicans* cells (Yin *et al*., 2004; Rodaki *et al*., 2006), thereby representing a ready source of organic nitrogen under conditions of nitrogen starvation.

The importance of ubiquitin-mediated protein turnover was further highlighted by our observation that the proteasomal components Pre1 and Pre3 are ubiquitinated in *C. albicans* (Table 2). Again this was consistent with findings in *S. cerevisiae*, where the proteasomal protein Pre5 is ubiquitinated (Kolkman *et al*., 2006).

Collectively, our data indicate that protein ubiquitination is highly dynamic in *C. albicans*, contributing to the regulation of key cellular processes. It is important to note that all of these processes underpin the virulence of this pathogen. Ubiquitination influences the growth and morphogenesis of *C. albicans*, which are fundamentally important for its virulence (Lo *et al*., 1997; Gow *et al*., 2002; Saville *et al*., 2003). Polyubiquitin is vital for robust stress adaptation in *C. albicans*, which is critical for pathogenicity (Alonso-Monge *et al*., 1999; Hwang *et al*., 2002; Smith *et al*., 2004; Hromatka *et al*., 2005; Enjalbert *et al*., 2006). Furthermore, effective metabolic adaptation in *C. albicans* is dependent upon polyubiquitin, and metabolic adaptation is central to host–fungus interactions during *C. albicans* infections (Lorenz and Fink, 2001; Lorenz *et al*., 2004; Brown, 2005; Fradin *et al*., 2005; Thewes *et al*., 2007; Zakikhany *et al*., 2007). It is not surprising therefore that our fifth conclusion is that polyubiquitin is important for the virulence of *C. albicans* in a mouse model of systemic candidiasis (Fig. 9). This study, and in particular our proteomic identification of ubiquitination targets, provides a platform for future studies to dissect the impact of specific target ubiquitination events upon the *C. albicans* cell and its virulence.

## Experimental procedures

### Strains, growth media and stress susceptibilities

*Candida albicans* strains (Table 1) were grown in YPD (Sherman, 1991) or synthetic complete medium lacking methionine and cysteine (Kaiser *et al*., 1994). The growth medium was supplemented with 2.5 mM methionine and cysteine for *MET3* promoter shut-off assays.

Susceptibilities to H_2_O_2_ (5 mM), NaCl (1 M), Calcofluor White (20 µg ml^−1^), Congo Red (100 µg ml^−1^), tunicamycin (4.73 mM), caspofungin (0.064 µg ml^−1^) and thermal stress (37 and 42°C) were tested using cells grown in YPD medium at 30°C to exponential phase (OD_600_ = 0.5). Cells were serially diluted in YPD, spotted onto YPD plates containing the appropriate supplements and examined after incubation for 48 h at 30°C (or at the temperatures specified). Experiments were done in duplicate and with independent mutants.

Metabolic adaptation phenotypes were examined by growing cells in YPD medium at 30°C to exponential phase (OD_600_ = 0.5). These cells were harvested, washed in 20 ml water and then resuspended at an OD_600_ of 0.5 in water (starvation), YNB-C (carbon starvation: containing ammonium sulphate but no carbon source or amino acids) or YNB-N (nitrogen starvation: containing 2% glucose but no amino acids or ammonium sulphate). These cell suspensions were then incubated with shaking at 30°C, and cell viability measured at various times thereafter by plating samples onto YPD to determine colony-forming units (cfu).

To test the impact of stresses upon ubiquitination levels, cells were examined after exposing them to the following stresses for 1 h in YPD: heat shock (30–42°C) by addition of an equal volume of pre-warmed YPD at 54°C; hyphal inducing conditions (37°C containing 10% fetal calf serum); Congo Red (100 µg ml^−1^); SDS (10%); Calcofluor White (100 µg ml^−1^); and H_2_O_2_ (50 mM).

### Strain construction

To generate conditional *C. albicans UBI4* mutants, one *UBI4* allele was placed under the control of the methionine/cysteine (Met/Cys) repressible *MET3* promoter (Care *et al*., 1999), and the other allele was deleted using the *lox-ARG4-lox* marker (Dennison *et al*., 2005). To achieve this, the *URA3-MET3p* cassette from plasmid pURA3-MET3 (Rodaki *et al*., 2006) was PCR amplified with *Pfu* Turbo (Promega; Southampton, UK) using the primers MetUS-F and MetUS-R (*Supporting information*– Primers) to generate a *URA3-MET3p-UBI4* cassette with 80 bp of homology to the *UBI4* 5′ upstream region and 80 bp of homology to the beginning of the *UBI4* open reading frame. Similarly, the *loxP-ARG4-loxP* sequence from plasmid pLAL (Dennison *et al*., 2005) was PCR amplified using primers LAL-F and LAL-R (*Supporting information*– Primers) to generate an *ubi4::ARG4* disruption cassette with 90 bp of flanking homology to the 5′ upstream and 3′ downstream regions of the *UBI4* gene. *C. albicans* strain BWP17 (Table 1) was then transformed with these cassettes as described previously (Gietz *et al*., 1995; Walther and Wendland, 2003), to generate the independent strains MLC03 and MLC12 (*MET3p-UBI4/UBI4*). These strains were then transformed with the *ubi4::ARG4* cassette to generate the independent conditional mutants MLC05 and MLC14 (*MET3p-UBI4/ubi4::ARG4*).

To generate *C. albicans ubi4* null mutants, the two *UBI4* alleles in the *C. albicans* strain BWP17 (*UBI4/UBI4*: Table 1) were inactivated sequentially with the *loxP-URA3-loxP* (LUL) and *loxP-ARG4-loxP* (LAL) markers (Dennison *et al*., 2005). This led to the construction of the independent *ubi4/ubi4* mutants, MLC40, MLC41 and MLC42 (Table 1). The *ubi4::LUL* and *ubi4::LAL* disruption cassettes, which were designed to delete the complete *UBI4* open reading frame, were generated by PCR amplification with the primers LAL-F, LAL-R and LUL-F, LUL-R (*Supporting information*– Primers). The *ubi4::LUL* cassette was transformed into *C. albicans*, transformants selected on the basis of their uridine protrophy, and correct integration confirmed by diagnostic PCR with primers UBI4d2-F and LULd-R (*Supporting information*– Primers). The resultant heterozygotes (*ubi4::URA3/UBI4*) were then transformed with the *ubi4::LAL* cassette, and accurate disruption of the remaining *UBI4* allele confirmed by diagnostic PCR using primers UBI4d2-F and LALd-R (*Supporting information*– Primers). This yielded the homozygous null mutants, MLC40, MLC41 and MLC42 (*ubi4::URA3/ubi4::ARG4*) (Table 1). The genotypes of all of mutants made in this study were confirmed both by PCR and Southern analysis using the ECL Direct Nucleic Acid Labelling and Detection System (GE Healthcare; Chalfont St Giles, Buckinghamshire, UK).

To generate a reintegrant control strain (*ubi4/ubi4/UBI4*), the *UBI4* locus was PCR amplified using the primers UBI4C-F and UBI4C-R (*Supporting information*– Primers), and ligated between the MluI and SalI sites of CIp30, a derivative of CIp10 (Murad *et al*., 2000; Dennison *et al*., 2005). The resulting CIp30-*UBI4* plasmid was digested with StuI and integrated at the *RPS10* locus in the *ubi4/ubi4* mutant (MLC41) to generate the control strain MLC61 (Table 1). To generate prototrophic control strains for the virulence studies, the empty CIp30 vector was integrated at the *RPS10* locus in MLC41 to generating MLC60, and CIp30 was transformed into BWP17 to generate MLC125 (Table 1).

### mRNA analyses

*UBI4* mRNA levels were measured by qRT-PCR. *C. albicans* cells were grown overnight at 30°C in YPD containing 2.5 mM Met/Cys. Cells were then reinoculated at OD_600_ = 0.1 into 50 ml of fresh YPD either containing or lacking Met/Cys, and regrown at 30°C to mid-log phase (OD_600_ = 0.5). Cells were harvested and frozen rapidly in liquid N_2_. RNA was extracted with Trizol (GibcoBRL, Grand Island, NY, USA) as described previously (Hauser *et al*., 1998), and RNA integrity assayed on an Agilent Bioanalyser 2100 (Stockport, UK). For qRT-PCR, samples were incubated at room temperature for 15 min in a 20 µl reaction mix containing 2 µg RNA, 2 µl DNase I buffer (Invitrogen; Paisley, UK), 1.5 µl DNase I and 1.5 µl RNase OUT (Invitrogen). cDNA was prepared using Superscript II (Invitrogen) as per the manufacturer's protocol. Real-time PCR was performed in triplicate in optical multiwall plate 384 using the LightCycler 480 Probes Master (Roche Applied Science; Burgess Hill, UK) as per the manufacturer's guidelines. Briefly, for the target transcripts *UBI4* and *ACT1*, probes were chosen using the ProbeFinder Software Version 2.45 (Roche, http://www.universalprobelibrary.com). PCR was performed in a 20 µl reaction containing 10 µl LightCycler 480 Probes Master Mix, 3 µl of 1:5 diluted cDNA, 0.2 µl of forward and reverse primers, 0.2 µl selective probe (Roche) and 6.4 µl water, PCR grade. Negative controls were performed using water instead of cDNA. Reactions were performed in a LightCycler 480 system (Roche Applied Science) using the following parameters: preincubation at 95°C for 5 min, 50 cycles of amplification at 95°C for 10 s and 60°C for 30 s, and a final cooling at 40°C for 1 min. Standard curves were prepared using four dilutions of the control, wild-type samples. *UBI4* mRNA levels were normalized against the internal *ACT1* mRNA control (in arbitrary units).

### Microscopy

Samples of exponentially growing *C. albicans* cells (OD_600_ = 0.5) were collected, fixed in 3.7% paraformaldehyde and examined by phase differential interference contrast microscopy. Cells were stained with 2 µg ml^−1^ CFW to visualize chitin. Nuclei were stained by overlaying samples with mounting media containing 1.5 µg ml^−1^ DAPI (Vector Laboratories, Peterborough, UK). All samples were examined by differential interference contrast and fluorescence microscopy using a Zeiss Axioplan 2 microscope. Images were recorded digitally using the Openlab system (Openlab v 4.04, Improvision, Coventry, UK) using a Hamamatsu C4742-95 digital camera (Hamamatsu Photonics, Hamamatsu, Japan).

### 1-D western analysis

Total soluble protein was extracted and subjected to Western blotting using published protocols (Smith *et al*., 2004). Briefly, cells were resuspended in 250 µl lysis buffer (0.1 M Tris-HCl, pH 8, 10% glycerol, 1 mM DTT, pepstatin A, protease inhibitor cocktail) and sheared with glass beads in a mini-bead beater (6 × 30 s with 1 min intervals on ice). Lysates were centrifuged at 13 000 r.p.m. for 10 min at 4°C. Proteins (15 µg) were separated by SDS-PAGE using the XCell *SureLock*™ Mini-Cell system (Invitrogen) with NuPAGE®Novex Bis-Tris 4–12% precast gels (Invitrogen) in NuPAGE® MOPS-SDS Running Buffer (Invitrogen) containing NuPAGE® Antioxidant (Invitrogen) as per the manufacturer's instructions. Proteins were transferred to Invitrolon™ PVDF Membranes (Invitrogen) in NuPAGE® Transfer Buffer containing methanol using the XCell II™ Blot Module (Invitrogen) as per the manufacturer's instructions. Following transfer, the membranes were rinsed in PBS and blocked in PBS-T + 5% BSA [PBS 0.1% Tween-20, 5% (w/v) BSA] for 1 h at room temperature. The membranes were then incubated overnight at 4°C in PBS-T + 5% BSA containing antibody. To detect ubiquitin, a 1:3000 dilution of anti-ubiquitin antibody was used (Stressgen, SPA-200, Exeter, UK). To detect Hsp90, a 1:10 000 dilution of anti-Hsp90 antibody was used (courtesy of Peter Piper) in PBS-T + 5% Milk [PBS 0.1% Tween-20, 5% (w/v) milk]. Membranes were incubated for 1 h at room temperature. Then an anti-rabbit HRP conjugated antibody was used at a 1:2000 dilution in PBS-T + 5% BSA for 1 h at room temperature. Membranes were washed in PBS-T and signals detected using an ECL Western blotting kit (Amersham, UK) as per the manufacturer's instructions. Signals were quantified using a Fusion FX7/SL Chemiluminescence and Fluorescence Combination Imaging System.

### Proteomics

*Candida albicans* protein extracts were prepared as described previously (Yin *et al*., 2004). Briefly, cells were disrupted with glass beads in 160 µl lysis solution [7.5 M urea, 2.5 M thio-urea, 1.25 mM EDTA, 1.75 µg ml^−1^ pepstatin A, 62.5 mM DTT, 25 mM Tris-Cl, pH 10.8 and 3.7 × protease inhibitor cocktail tablets (Roche, Lewes, UK)] using a bead beater. After cell disruption, proteins were further solubilized by adding 40 µl of Lysis 2 [20% w/v CHAPS, 50% v/v glycerol, 10% v/v carrier ampholyte (pH 4–7)], and incubated on ice for 1 h. After repeated bead beating, cell debris were removed by centrifugation (20 min, 13 000 r.p.m., 4°C) and protein extracts were stored at −20°C.

Two dimensional gel electrophoresis was performed as described previously (Cash and Argo, 2009). After rehydration, IPG strips (pH 4–7 linear) were used to separate proteins on a Multiphor II (75–100 µg) in the first dimension according to the following protocol: 200 V for 1 min, 3500 V for 90 min, 3500 V for 65 min. After isoelectric focusing, IPG strips were equilibrated in two steps: (i) 50 mM Tris-Cl (pH 6.8), 6 M urea, 30% v/w glycerol, 2% w/w SDS, 62.5 mM DTT for 25 min; and (ii) 50 mM Tris-Cl (pH 6.8), 6 M urea, 30% v/w glycerol, 2% w/w SDS, 2.5% w/v iodoacetamide for 25 min. Proteins were then separated in the second dimension on precast 4–12% SDS polyacrylamide gels (Invitrogen) using MOPS buffer at first with 100 V per gel for 30 min, followed by 200 V per gel for a further 90 min. Gels were fixed (2% H_3_PO_4_, 50% ethanol) overnight, rinsed three times with water and equilibrated [1.3 M (NH_4_)_2_SO_4_, 2% w/w H_3_PO_4_, 34% w/v methanol] for 60 min, before staining with colloidal Coomassie blue G250 (0.67 g l^−1^). Equivalent 2-D gels were subjected to Western blotting to detect ubiquitin-conjugated proteins, as described above for 1-D gels. Independent biological replicates were performed for all experiments and only reproducible observations are reported.

Ubiquitinated proteins were selected by aligning the Western blots with the corresponding Coomassie-strained gels, and these proteins identified by liquid chromatography – tandem mass spectrometry (LC-MS/MS). The corresponding spots (1.2 mm diameter) were cut from gels and transferred to 96-well microtitre plates using an Investigator ProPic robotic workstation (Genomic Solutions; Huntingdon, UK). Proteins in gel plugs were digested with trypsin (Promega, UK) using an Investigator ProGest robotic workstation (Genomic Solutions). Peptides were extracted, dried in a SpeedVac SC110A (Savant Instruments) and dissolved in 0.1% formic acid for LC-MS/MS. The LC-MS system comprised an UltiMate 3000 LC system (Dionex) coupled to an HCTultra ion trap mass spectrometer with ESI source fitted with a low-flow nebulizer (Bruker Daltonics). Peptides were separated on a PepSwift monolithic capillary column (Dionex) at 2 µl min^−1^ using a linear gradient of acetonitrile. Eluent A was 3% acetonitrile in 0.05% formic acid and Eluent B was 80% acetonitrile in 0.04% formic acid. The gradient was 3–45% Eluent B over 12 min at a flow rate of 2 µl min^−1^.

Tandem mass spectra were acquired in data-dependent AutoMS(2) mode using the following parameters: MS scan range = 300–1500 m/z; averages = 3, max. no. of precursors = 3; MS(2) scan range = 100–2200 m/z, averages = 2; active exclusion on (max. spectra = 2, release after 1 min). Peptide peaks were detected and deconvoluted automatically using DataAnalysis software (Bruker). Mass lists were used as input for Mascot MS/MS Ions searches using Mascot Server version 2.2 (Matrix Science, London, UK). Our in-house protein sequence database (containing 6166 sequences) was built from the flat file <CALBI_prot.txt> downloaded from the CandidaDB web server ftp://ftp.pasteur.fr/pub/GenomeDB/CandidaDB/FlatFiles. Protein annotations were taken from the Candida Genome Database (http://www.candidagenome.org/) and CandidaDB (http://genolist.pasteur.fr/CandidaDB/). Potential ubiquitination sites were predicted using the program UbPred (Radivojac *et al*., 2010).

Our proteomics dataset can be accessed via the PRoteomics IDEntifications database (PRIDE) data repository at the European Bioinformatics Institute (http://www.ebi.ac.uk/pride) (Vizcaino *et al*., 2010) using Accession Number 13 697.

### *C. albicans* virulence assays

All animal experimentation conformed to the requirements of the UK Home Office legislation and of the Ethical Review Committee of the University of Aberdeen.

The virulence of *C. albicans* strains MLC125, MLC60 and MLC61 (Table 1) was assessed in the mouse intravenous challenge model (MacCallum *et al*., 2010). Female BALB/c mice (6–8 weeks old; Charles River, UK) were infected intravenously with a saline suspension of *C. albicans* cells (4 × 10^4^ cfu g^−1^ mouse body weight in a 100 µl volume; six mice per *C. albicans* strain), prepared from cells grown for 18–24 h in NGY medium at 30°C (MacCallum and Odds, 2005). At 72 h post infection, mice were weighed, humanely terminated, the kidneys removed aseptically and renal fungal burdens determined. Infection outcome scores were calculated on the basis of weight change and renal fungal burdens, as described previously (MacCallum *et al*., 2010). For virulence assays, kidney burdens and infection outcome scores were compared by the Kruskall–Wallis and Mann–Whitney *U*-tests. Statistical analyses were carried out using PASW statistics 18.0.
