# Supplementary material for: Molecular and proteomic analyses highlight the importance of ubiquitination for the stress resistance, metabolic adaptation, morphogenetic regulation and virulence of Candida albicans
Source: Mol Microbiol. 2011 Mar;79(6):1574–93. doi: 10.1111/j.1365-2958.2011.07542.x (PMC3084552; doi:10.1111/j.1365-2958.2011.07542.x)
Supplement: Supplementary file 1 [file mmi0079-1574-SD1.pdf]

Summary of Supplementary Data

Sheet	Title	Description
1	Summary	Summary of Supplementary Data
2	Ubiquitination Targets	List of ubiquitination targets in *C. albicans*
3	MET3p-UBI4 control	Effects of stress upon *C. albicans MET3p-UBI4/ubi4* in the absence of methionine and cysteine
4	Ponceau S Stain	Ponceau S stain of *UBI4/UBI4, UBI4/ubi4 and ubi4/ubi4* to control loading
5	Primers	List of PCR primers used in this study

Identification of probable ubiquitination targets in *C. albicans*

Sample Ref	Accession Number (CandidaDB)	Accession Number (CGD)	Protein Name	Mol. Wt. (Da)	pI	Mascot Score	Peptides Matched	Sequence Coverage	ELDP	Function	Ubiquitinated in *S. cerevisiae*?	Predicted Ubiquitination Site(s)	Ubiquitination detected in absence of stress?	Ubiquitination upregulated in response to stress?
Constitutively ubiquitinated														
1	Ca2937	orf19.3325.3	RPS21 B	8807	8.85	372	4	48%	2	Similar to ribosomal protein S21	No	Medium confidence, position 5	Yes	Downregulated
2	Ca1691	orf19.3651	PGK1	45266	6.07	2080	37	84%	12	Phosphoglycerate kinase	Yes	Medium confidence, position 6; low confidence, position 296	Yes	No
3	Ca5180	orf19.4618	FBA1	39362	5.69	2000	24	79%	9	Putative fructose-bisphosphate aldolase	Yes	Medium confidence, position 72 and 192; low confidence, position 77	Yes	No
4	Ca2939	orf19.3324	TIF1	44742	5.22	1397	26	72%	15	Translation initiation factor	Yes	Low confidence, position 24	Yes	No
	Ca0824	orf19.691	GPD2	41174	5.21	313	9	33%	7	Glycerol-3-phosphate dehydrogenase	Yes	Low confidence, position 274 and 318	Yes	No
Increased ubiquitination in response to heat														
5	Ca3874	orf19.395	ENO1	47202	5.54	204	7	23%	7	Enolase	Yes	High confidence, position 273; medium confidence, position 265; low confidence, position 432	Yes	Heat
6	Ca4389	orf19.3123	RPT5	47814	5.25	1059	21	60%	13	Regulatory particle triphosphatase	Yes	Medium confidence, position 55, 63, 163, 170, 284 and 290; low confidence, position 35 and 92	Yes	Heat
7	Ca3924	orf19.5112	TKL1	73841	5.48	1036	23	45%	10	Putative transketolase	Yes	Low confidence, position 351	No	Heat
8	Ca2895	orf19.2461	PRN4	37072	5.31	496	16	47%	3	Putative pirin by homology	No	High confidence, position 315; medium confidence, position 234; low confidence, position 240, 320 and 327	No	Heat
9	Ca5239	orf19.5024	GND1	57159	6.14	478	19	38%	13	Putative 6-phosphogluconate dehydrogenase	Yes	Medium confidence, position 6; low confidence, position 151 and 388	No	Heat
	Ca2011	orf19.3087	UBI3	17485	9.86	42	1	14%	0	Functional homologue of *S. cerevisiae* RPS31	No	Medium confidence, position 105	No	Heat
10	Ca0210	orf19.5525	IPF4328	37783	5.53	417	11	42%	9	Putative NADP(H) oxidoreductase	No	Medium confidence, position 285; low confidence, position 268 and 344	No	Heat
Increased ubiquitination in response to H_2O_2														
11	Ca4261	orf19.550	PDX3	28773	6.02	356	13	68%	1	Pyridoxine (pyridoxamine) phosphate oxidase	Yes	Medium confidence, position 22 and 58	No	H_2O_2
12	Ca2675	orf19.5493	GSP1	24470	6.53	146	5	35%	5	Small RAN G-protein	Yes	Medium confidence, position 193	No	H_2O_2
	Ca5932	orf19.6771	UBI4	25776	7.76	53	1	10%	1	Ubiquitin precursor (polyubiquitin)	Yes	Low confidence, postion 33, 48, 63, 139, 179 and 215	No	H_2O_2
13	Ca1673	orf19.3612	PST2	21714	6.51	206	7	52%	7	Putative NADH:quinone oxidoreductase	Yes	None	No	H_2O_2

	Ca5037	orf19.6991	PRE3	23629	6.42	155	7	32%	3	**B**eta-1 subunit of proteasome	No	Low confidence, postion 12	No	H_2O_2
14	Ca4791	orf19.4025	PRE1	22119	6.43	273	11	38%	4	Beta 4 subunit of the 20S proteasome (*S. cerevisiae*)	No	Low confidence, position 19	No	H_2O_2
	Ca4589	orf19.6906	ASC1	23619	6.3	85	5	27%	3	40S ribosomal subunit protein	Yes	None	No	H_2O_2
	Ca5932	orf19.6771	UBI4	25776	7.76	76	3	24%	1	Ubiquitin precursor (polyubiquitin)	Yes		No	H_2O_2
Increased ubiquitination in response to heat and H_2O_2														
15	CA3534	orf19.6367	SSB1	66580	5.25	2786	35	67%	22	Putative HSP70 family heat shock protein	Yes	High confidence, position 528 and 545; medium confidence, position 521, 530, 543 and 597; low confidence, position 191, 314 and 393	No	Heat and H_2O_2
16	Ca1246	orf19.18	IMH3	40055	6.67	1879	25	74%	8	Inosine monophosphate (IMP) dehydrogenase	No	Medium confidence, position 7; low confidence, position 24, 175, 230 and 517	No	Heat and H_2O_2
17	Ca3546	orf19.6385	ACO1	84625	5.96	1669	38	52%	22	Aconitase	No	Medium confidence, position 25.	No	Heat and H_2O_2
18	Ca2858	orf19.1064	ACS2	74215	5.73	1300	32	54%	20	Probable acetyl-CoA synthetase	Yes	Medium confidence, position 32 and 596; low confidence, position 17 and 583	No	Heat and H_2O_2
19	Ca2470	orf19.2871	SDH12	70930	6.03	790	21	47%	11	Flavoprotein subunit of succinate dehydrogenase	No	Medium confidence, position 480; low confidence, position 417, 492 and 500	No	Heat and H_2O_2

Effects of stress upon *C. albicans MET3p-UBI4/ubi4* in the absence of methionine and cysteine

NO Met/Cys control

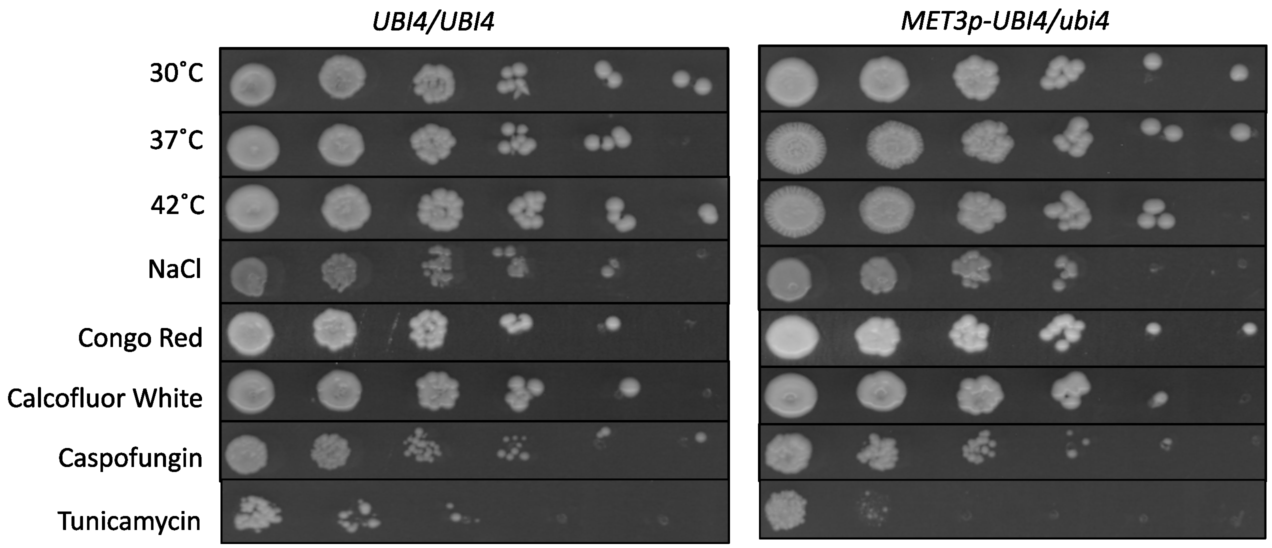

PLUS Met/Cys (Figure 4B)

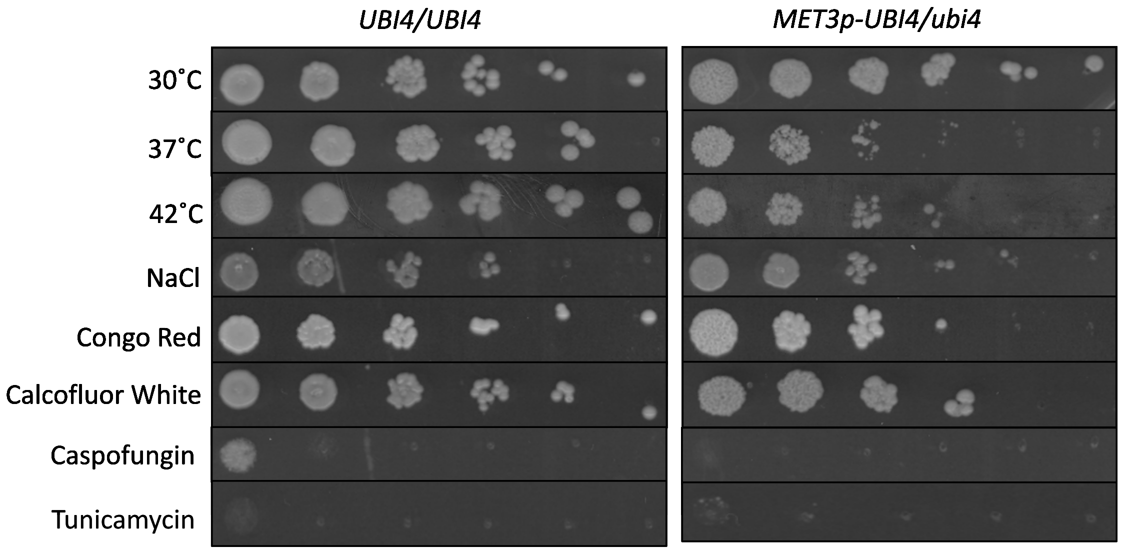

Sensitivity of a MET3p-UBI4/ubi4 conditional mutant to stresses. Serial dilutions were spotted onto SC plates containing the appropriate stress and containing 2.5 mM methionine and cysteine: UBI4/UBI4 (BWP17); MET3p-UBI4/ubi4 (MLC05). These strains grew at similar rates on control plates that lacked methionine and cysteine (Above).

Ponceau S staining to demonstrate even loading

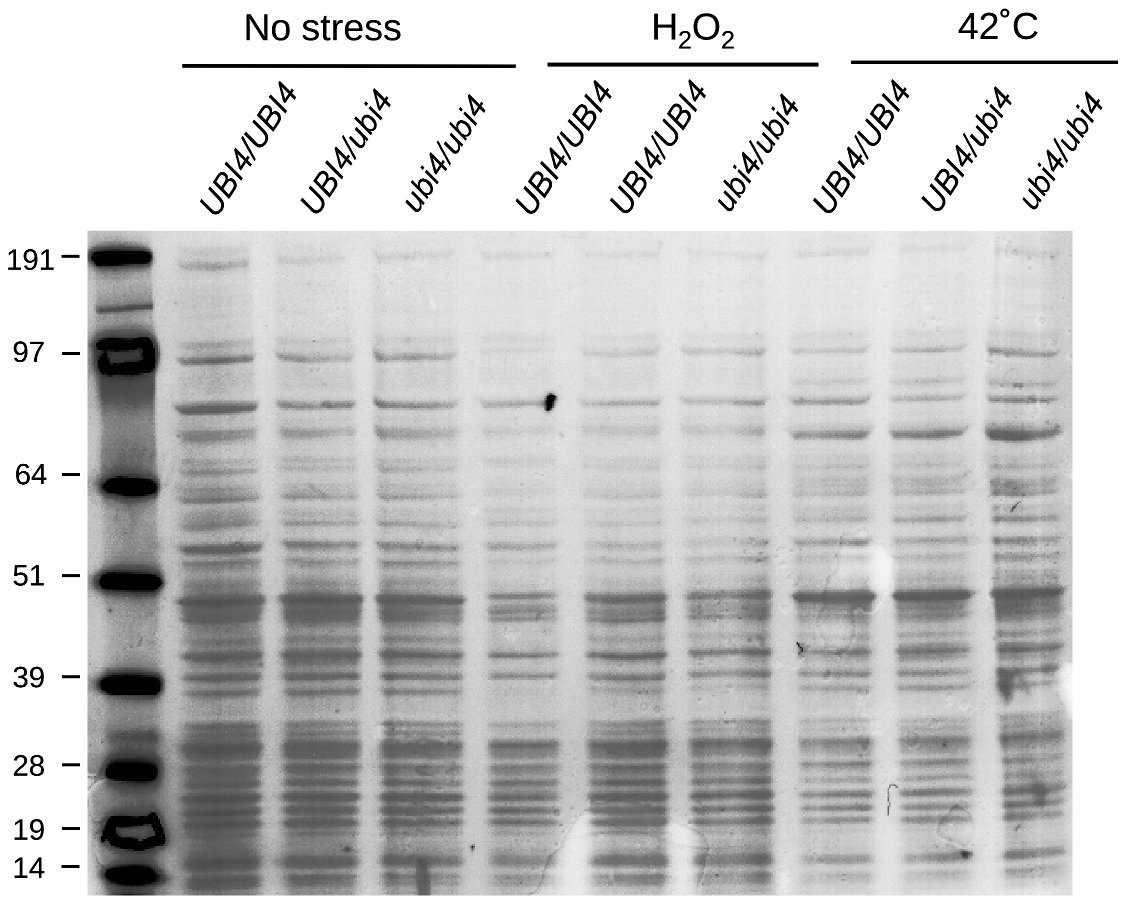

Oligonucleotides used in this study

Primer	Sequence (5' to 3')	Application
UBI4d-F	CCTAGTTGCCTTAACGACAG	Diagnosis of *UBI4* allele
UBI4d2-F	GCGACATTAAAATAGGAGAGGC	Diagnosis of *UBI4* , *ARG4* and *URA3* alleles
UBI4d-R	GTCAGACAAGGTTCTGCC	Diagnosis of *UBI4* and *URA3-MET3-UBI4* alleles
UBI4d2-R	CTTGAGAAGACATAAGTAAGG	Diagnosis of *UBI4, ARG4, URA3* and *URA3-MET3-UBI4* alleles
MET3p-F	ATTGCTGTGGATCACGTGC	Diagnosis of *URA3-MET3-UBI4* allele
LALd-R	GCCCATCTAATAGGTTGAGC	Diagnosis of *UBI4::ARG4* allele
LULd-R	GTATGGGGTTGTTGCTCAGG	Diagnosis of *UBI4::URA3* allele
MetUS-F	CCTCGTTCATTCTAATTCAATACAAGTTTGTATTGTTTATACCATGTTTAT<br>ACAAACGGGTTTAATTTATAATTACGGTAGCCGGCCGACTTGGCCAAG<br>CCTAGATC	Amplification of *URA3-MET3-UBI4* cassette
MetUS-R	GGATTTGACGTTATCGATGGTGTCAGAAGATTCGACTTCTAAGGTAATG<br>GTTTTACCAGTCAAAGTTTTAACGAAAATTTGCATTGGGGAGGGTATTT<br>ACTTTTAAATA	Amplification of *URA3-MET3-UBI4* cassette
LAL-F	CCTTACGTACAGCACACACATACCACTGTCGTGCACTGACCAACAACA<br>ATGCGGTGTTAATCGATAACCAAAAGATTATAAATAGGGGGTGGAAGG<br>TCGCCCCAGGGTTTTCCCAGTCACG	Amplification of *UBI4::ARG4* cassette
LAL-R	GCAGCAATGATTATAAAAAATTAGAAACCACCTCTCAATCTCAAGACCA<br>AGTGCAAGGTAGATTCTTTTTGGATGTTGTAGTCAGACAAGGTTCTGCC<br>ACTAAAGGGAACAAAAGC	Amplification of *UBI4::ARG4* cassette
LUL-F	CATACATTGGGTAGGTCATTCATAACAATTGATAGATGCAAGCTAATTG<br>GAATGAAAAATCCATCTTGTATCAAAACCCTTTGTTCCTCATAGTTAATC<br>CGCCAGGGTTTTCCCAGTCACG	Amplification of *UBI4::URA3* cassette
LUL-R	CTTTATAGAGTTTATAAATGTCAGTTTAAGCTGAAAAAAAATTATTATAG<br>TACAAATGTAAGATACTCTCATAATCAATGGGACCTGAATTGGACCCAC<br>TAAAGGGAACAAAAGC	Amplification of *UBI4::URA3* cassette
UBI4S-F	CGTCAACTAACATATATATAC	Amplification of *UBI4* probe for Southern analysis
UBI4S-R	GCCTAGTCTATAAAACAAAAC	Amplification of *UBI4* probe for Southern analysis
LALS-F	GGTAGATCAAGAAATGATC	Amplification of *ARG4* probe for Southern analysis
LALS-R	CAATCAGTAAAGTAGAAATC	Amplification of *ARG4* probe for Southern analysis
LULS-F	CCTATAGTGAGAGAGCAG	Amplification of *URA3* probe for Southern analysis
LULS-R	GGATCTCTTCCTTTACC	Amplification of *URA3* probe for Southern analysis
ACT1-F	ACCACCGGTATTGTTTTGGA	Real time RT-PCR of *ACT1* transcript levels
ACT1-R	AGCGTAAATTGGAACAACGTG	Real time RT-PCR of A*CT1* transcript levels
ACT1-P	ACCACCGGTATTGTTTTGGATTCTGGTGATGGTGTTACTCACGTTGTTC<br>CAATTTACGCT	Probe for real time RT-PCR of *ACT1* transcript levels
UBI4q-F	CGTCAAATCCAAGATCCAAGA	Real time RT-PCR of *UBI4* transcript levels
UBI4q-R	CGGCGAAAATCAATCTTTGT	Real time RT-PCR of *UBI4* transcript levels
UBI4q-P	CCATCGATAACGTCAAATCCAAGATCCAAGACAAAGAAGGTATTCCACC<br>AGACCAACAAAGATTGATTTTCGCCGGTAAACAATT	Probe for real time RT-PCR of *UBI4* transcript levels
UBI4-F	GAACAGATATGTTGGTTCCAGGATTGAAAAG	Amplification of *UBI4*
UBI4-R	CCTTATTTGTAGACTCGGGTAATAGC	Amplification of *UBI4*
UBI4C-F	TATCGAATTCACGCGTGACTCAGATCGATAAGCGAACATCACAGC	Amplification of *UBI4* for cloning
UBI4C-R	GGTACCTGGAGTCGACCAATGTGAGGACTGGCTAACTTTAATAGG	Amplification of *UBI4* for cloning
